# Controllable synthesis of conjugated microporous polymer films for ultrasensitive detection of chemical warfare agents

**DOI:** 10.1038/s41467-022-32878-w

**Published:** 2022-09-03

**Authors:** Wanqi Mo, Zihao Zhu, Fanwei Kong, Xiaobai Li, Yu Chen, Huaqian Liu, Zhiyong Cheng, Hongwei Ma, Bin Li

**Affiliations:** 1 Key Laboratory of Forest Plant Ecology, Ministry of Education, Engineering Research Center of Forest Bio-Preparation, College of Chemistry, Chemical Engineering and Resource Utilization, Northeast Forestry University, Harbin, 150040 P. R. China; 2 Post-doctoral Mobile Research Station of Forestry Engineering, Northeast Forestry University, Harbin, 150040 P. R. China

**Keywords:** Sensors, Conjugated polymers, Sensors and biosensors

## Abstract

Nerve agents, one of the most toxic chemical warfare agents, seriously threaten human life and public security. The high toxicity of nerve agents makes the development of fluorescence sensors with suitable limit of detection challenging. Here, we propose a sensor design based on a conjugated microporous polymer film for the detection of diethyl chlorophosphate, a substitute of Sarin, with low detection limit of 2.5 ppt. This is due to the synergy of the susceptible on-off effect of hybridization and de-hybridization of hybrid local and charge transfer (HLCT) materials and the microporous structure of CMP films facilitating the inward diffusion of DCP vapors, and the extended π-conjugated structure. This strategy provides a new idea for the future development of gas sensors. In addition, a portable sensor is successfully integrated based on TCzP-CMP films that enables wireless, remote, ultrasensitive, and real-time detection of DCP vapors.

## Abstract (web summary)

The high toxicity of nerve agents makes the development of fluorescence sensors with suitable limit of detection challenging. Here, the authors propose a sensor design based on a conjugated microporous polymer film for the detection of diethyl chlorophosphate, a substitute of Sarin, with low detection limit of 2.5 ppt.

## Introduction

Organophosphorus nerve agents are an extremely toxic class of chemical warfare agents that can rapidly destroy the transmission of human nerve impulses due to their potent inhibition of the hydrolytic enzyme of neurotransmitter acetylcholine, causing paralysis of the central nervous system and eventually death^1,2^. The development of efficient and reliable detection technologies for nerve agents, as one countermeasure of paramount importance, is therefore becoming an urgent topic, but also an arduous task because of their colorless, odorless, and volatile nature^3^. Furthermore, owing to the strong lethality, access to nerve agents is tightly restricted. Less poisonous simulants, e.g., diethyl chlorophosphate (DCP) is usually employed as substitutes of Sarin to facilitate the advance of detection technologies^4,5^. Of various detection methods exploited, such as mass spectroscopy^6^, ion mobility spectrometry^7^, electrochemical sensors^8^, and biosensors, fluorescent detection^9^ has galvanized interest due to numerous appealing virtues that include good portability, simple operation, high sensitivity, fast response, low cost, and real-time monitoring^10,11^.

Inspired by the mechanism that nerve agents inhibit the acetylcholinesterase (AChE) via reactions of their electrophilic phosphorus with the nucleophilic hydroxyl groups of AChE, fluorescent sensing materials with nucleophilic functional groups, including organic metal^12^, *N*-based aromatic heterocycle^13^, *N*- or *O*-containing compounds^14^, have been studied during the last decade. Although fluorescent sensors are normally more sensitive than other detection strategies attributed to the fast photoinduced electron transfer (PET)^15^ or fluorescence resonance energy transfer (FRET)^16^ caused by phosphorylation resulting in fast changes of luminescence characteristic, even higher sensitivity is practically demanded because of the ultralow lethal dose (7 ppb) of Sarin. Recently, we proposed a new type of nucleophilic fluorescent molecule with a hybrid local and charge transfer (HLCT) excited state, which has an impressive sensitivity for DCP vapors with a limit of detection (LOD) of 0.15 ppb, much lower than the lethal dose, and a fast response of less than 1 s in saturated DCP vapors^17^. This is due to its peculiar hybrid state, which is highly susceptible to the attack of DCP, leading to a more rapid and effective fluorescent quenching response because of de-hybridization. Nevertheless, fluorescent small molecule materials for DCP frequently suffer from a common problem of serious photobleaching in sensor integration because of the unoptimistic anti-photobleaching ability, restricting their further applications^16^.

In contrast, polymer fluorescent films possess preferable photostability. While, for solid-state sensing films, to realize efficient detection for trace amounts of nerve-agent vapors, the assistance of favorable gas capture capacity is desired. In a recent review, analyte diffusion into films is highlighted as a critical factor in the design of fast and responsive sensing systems^18^. Conjugated microporous polymers (CMPs) as a unique class of polymers featured by the extended π-conjugated system and inherent microporous structure have currently aroused increasing attention in a variety of research fields, for instance, organic optoelectronics^19^, photocatalytic^20^, gas adsorption, and storage^21^, as well as fluorescent sensing^22^. The micropores of CMPs are demonstrated to be beneficial to the inward diffusion of analytes, e.g., gas molecules and metal ions. Further, π-skeletons facilitate the exciton delocalization that is better able to amplify the fluorescence response signals and thus enhance sensing sensitivity, compared with small molecular materials^23^. However, the film processability of CMPs limited by their poor solubility is the bottleneck issue in their practical applications^24^. Indeed, most studied fluorescence CMPs are insoluble solids that are unsuitable for sensor integration^25^. As is evident, challenges remain in developing a highly sensitive fluorescent material that has a desirable combination of good photostability, easy analyte diffusion, and facile processability (i.e., ease of sensor implementation) for nerve-agent detection^18^.

In our previous work, high-quality CMP fluorescence films were successfully fabricated by a simple electropolymerization (EP) method and the versatile sensing platforms were constructed^26,27^. Building on this important result, herein, we propose a new strategy to access ultrasensitive and photo-stable films for DCP vapors sensing. Two HLCT molecules (TCz, TCzP) as the precursors with good electrochemical activity and different π-conjugated structures are designed and synthesized (Supplementary Fig. 1). The resulted TCz-CMP and TCzP-CMP films have different pore sizes, which directly affect the diffusion of DCP in the CMP films. It is found that two CMP films are ultrasensitive to DCP vapors, and the LOD of TCz-CMP (thickness 45 nm) and TCzP-CMP (thickness 250 nm) can be determined as 21 ppt and 2.5 ppt of DCP vapors, respectively, which are much lower than that of the corresponding monomers. This is one of the best LOD value of DCP vapors among fluorescent sensing materials reported so far. This result is due to the efficient synergy of the excellent fluorescence response mechanism of HLCT, the extended π-conjugated structure, and the inward diffusion of DCP vapors. As a result, a portable sensor is successfully integrated based on TCzP-CMP films that enable wireless, remote, ultrasensitive, and real-time detection of DCP vapors. Meanwhile, TCzP-CMP has a DCP adsorption capacity of to 936 mg/g, which is ~3 times that of activated carbon, indicating a potential of combining nerve-agent detection with military protection.

## Results

### Optical properties of polymer precursors

**Fig. 1:**
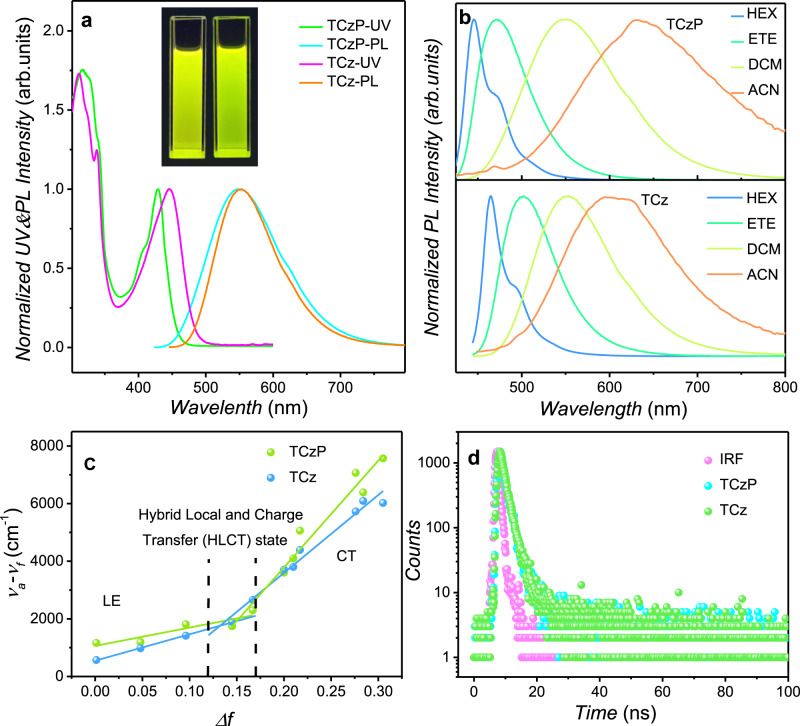
Optical properties of polymer precursors. **a** The UV-Vis and PL spectra of TCz and TCzP in DCM. Inset: the photo shows the fluorescence of TCzP (left) and TCz (right) in DCM solution excited by a wavelength of 365 nm. **b** The solvatochromic effects of TCz and TCzP in the increasing polarity solvent (HEX, ETE, DCM and ACN). **c** Solvatochromic Lippert-Mataga models of TCz and TCzP. **d** Transient PL spectra of TCz and TCzP in isopropyl ether.

Herein, two fluorescence materials TCz and TCzP composed of carbazole as the donor and dibenzo [a, c] phenazine (DPPZ) as the acceptor were designed (Supplementary Figs. 1–7). Compared with TCz, an additional phenyl ring is inserted between carbazole and DPPZ group for TCzP, the design strategy for extended π-conjugated structure is to increase locally emissive (LE) component in the excited state and to optimize the size of the micropores in CMP obtained by EP. Then, the ultraviolet-visible (UV-Vis) absorption and photoluminescence (PL) properties of TCz and TCzP were investigated to understand the basic photophysical properties (Fig. 1a). TCzP and TCz show almost the same absorption peaks about 317 nm, which are ascribed to the π-π* of carbazole. As a comparison, the TCzP shows a narrower absorption band around 429 nm than that of TCz (445 nm), indicating a more π-π* like character. For the PL spectra, TCz and TCzP give a yellow emission with λ_max_ at 553 nm and 548 nm, respectively, and the abnormal 5 nm blue-shift of TCzP compared to TCz in PL spectrum can be attributed to the enhanced LE component in the emissive state of TCzP^28^.

To better understand the excited state properties, the solvatochromic effect of TCzP and TCz were further investigated. The PL spectra of TCzP and TCz show significant red-shift as the solvent polarity increases, which is the typical feature of the charge transfer (CT) state. Moreover, the PL peaks of TCzP exhibit a larger red-shift (185 nm) than TCz (130 nm) from hexane (HEX), ethyl ether (ETE), dichloromethane (DCM) to acetonitrile (ACN), indicating that TCzP is more susceptible to external stimulation than TCz. Obviously, these features are expected to help construct rapid and sensitive sensors (Fig. 1b). It is well-known that the photoluminescence quantum yield (PLQY) of CT molecules should be decreased with the increase in the solvent polarity because the high-polarity solvents could induce a stronger CT state and result in low PLQY. Unlike the common CT molecules, the two molecules show greatly enhanced PLQY with the increase of the solvent polarity, which is the result of the HLCT formation. Nevertheless, the CT-dominated HLCT state could occur in the large-polarity solvents, resulting in a decrease in PLQY (Supplementary Table 1, Supplementary Note 1)^29^.

In order to further clearly reveal the singlet state properties of TCz and TCzP, the linear relationships of the slope of Stokes shift (*ν*_a_–*ν*_f_) verse solvent polarity (*f*) using the Lippert-Mataga equation (Supplementary Equation 3, Supplementary Note 2) were carried out (Fig. 1c and Supplementary Table 2)^30,31^. In fact, two segmental fitting lines in TCz and TCzP represent the existence of two excited states. In low-polarity solvents (*f* < 0.12), the lesser slope fitting lines with the small dipole moments (*µ*_e_) are attributed to the LE state, while in high-polarity solvents (*f* > 0.16), the higher slope fitting lines with large *µ*_e_ belong to the CT state. As a comparison, TCzP has a larger *µ*_e_ (37.11 D) related to TCz (28.72 D), resulting in that TCzP showing a more obvious red-shift in the solvatochromic shift. In medium-polarity solvents, the energy levels of intrinsic LE and CT excited states are relatively close, and the coupling and crossing between the LE and CT states promote the formation of the HLCT state^32^, which is consistent with the solvatochromic results. In addition, the lifetime decay curves of TCz and TCzP in a medium-polarity solvent show a single exponential lifetime of 1.38 ns and 1.37 ns, respectively, indicating that the HLCT excited state is a hybrid state, rather than a single mixed state of LE and CT (Fig. 1d).

### Sensing performance and sensing mechanism of polymer precursors

A recent review proposed that *N*-heterocyclic-based nerve-agent fluorescence sensors should consider the influence of acid impurities produced by the hydrolysis of DCP on detection performance^18,33^. Therefore, the detection performance of TCz (thickness 4 nm) and TCzP (thickness 6 nm) spin-coated films for acid-free DCP vapors under N_2_ was first investigated, and their LODs were calculated to be 69 ppb and 6.6 ppb, respectively (Supplementary Fig. 8, Supplementary Tables 3, 4 and Supplementary Note 3). These performances are attributed to the rapid de-hybridization of HLCT excited states. Considering that the hydrolysis of DCP is inevitable in the actual detection, and it is convenient to compare the detection performance of HLCT excited state materials with other fluorescent materials, the fluorescence response of TCz (thickness 4 nm) and TCzP (thickness 6 nm) spin-coated films to acid-containing DCP vapors was performed. The DCP used for the detection was obtained using the common preparation methods (acid-containing, Supplementary Note 4). As shown in Supplementary Fig. 9, the LODs of TCz and TCzP for DCP vapor are 1.9 ppb and 1.0 ppb, respectively. They had a higher sensitivity to DCP vapors (acid-containing) than DCP vapors (acid-free). In order to further illustrate the influence of HCl on the detection performance of TCz and TCzP to DCP, the fluorescent responses of TCz and TCzP to HCl were tested, and their LODs were calculated to be 84 ppt and 140 ppt, respectively (Supplementary Fig. 10). To further confirm the detection mechanism of TCz and TCzP to DCP, the titration experiments of the ^1^H NMR were performed. Upon addition of DCP to TCz and TCzP in CDCl_3_, respectively, the chemical shifts of the protons on the DPPZ move downfield, while no significant change for the other groups (Supplementary Fig. 11). This result may be attributed to the nucleophilic substitution reaction between TCz or TCzP and DCP, and the formed intermediate quickly undergoes a hydrolysis reaction with trace water, resulting in the protonation of the N atom in the DPPZ. Furthermore, ^31^P NMR experiments were performed to reveal this hydrolysis process (Supplementary Fig. 12). Upon addition of TCz to DCP in CDCl_3_, three new chemical shift peaks appear at 1.527 ppm, 0.630 ppm, and −12.599 ppm, which correspond to diethyl phosphate, the intermediate and tetraethyl pyrophosphate (TEPP), respectively. According to previous reports^34^, the TEPP is obtained through the nucleophilic substitution between diethyl phosphate and DCP, and diethyl phosphate is produced through the hydrolysis of intermediates with water. Meanwhile, it is found that the quenched TCz or TCzP by acid-free DCP vapor or HCl vapors have an obvious fluorescence recovery under N_2_ blowing, indicating that the fluorescence response is reversible to a certain extent (Supplementary Fig. 13). After further research, a reasonable detection mechanism is proposed (Supplementary Figs. 14–17, Supplementary Note 5).

### Calculated excited state properties

**Fig. 2:**
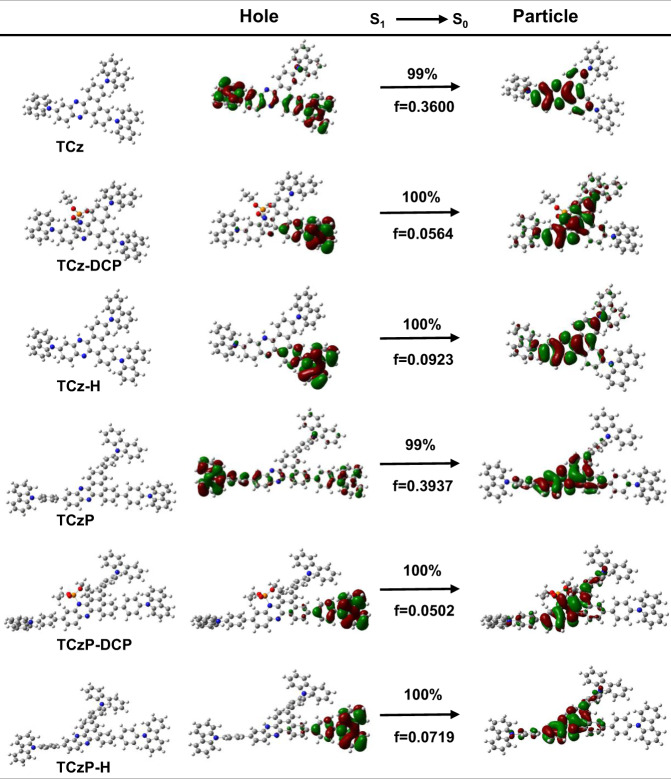
Calculated excited state properties. The NTO analysis of S_1_→S_0_ transition for TCz and TCzP before and after exposure to DCP.

The quenching processes of TCz and TCzP to DCP were confirmed by time-dependent density functional theory (TDDFT) calculations. Both the energy levels of the intermediate and protonation of two fluorescence molecules exhibits obviously decrease accompanied with quenching (Supplementary Fig. 18, Supplementary Note 6). The S_1_ energy level difference of TCz (1.45 eV) before and after protonation is significantly smaller than that of TCzP (1.99 eV), indicating that TCzP is more sensitive to DCP than TCz. In addition, the energy level and the oscillator strength (*f*) of phosphorylated intermediates is lower than that of protonated products, which makes it possible to conclude that the two fluorescence molecules are more inclined to generate phosphorylated intermediates and then form the protonated product. Then, the essence of their excited state quenching processes was also revealed by natural transition orbit (NTO) in terms of S_1_ excited states (Fig. 2). Before protonation, the S_1_ transition of the two small fluorescence molecules is mainly the LE-dominated HLCT excited state, which is ascribed to S_1_ transition of DPPZ. After protonation, the hole-electron separation is intensified, and there is almost no overlapping hole-electron distribution. Meanwhile, the oscillator strength is reduced and the vertical electron effect of the TCz and TCzP is forbidden. The results show that the uniform HLCT state is destroyed and forming a strong non-emissive CT state, which corresponds to the new absorption peak at 596 nm (TCz) and 580 nm (TCzP) in the absorption spectra, respectively (Supplementary Fig. 19). In this case, the uniform HLCT state of the two fluorescence molecules becomes a separate LE state and CT state, leading to a significant fluorescence quenching. However, the two fluorescence films faced serious photobleaching problems, which shorten the working life of these films and are not suitable for device integration.

### The preparation of CMP films and pore size analysis

**Fig. 3:**
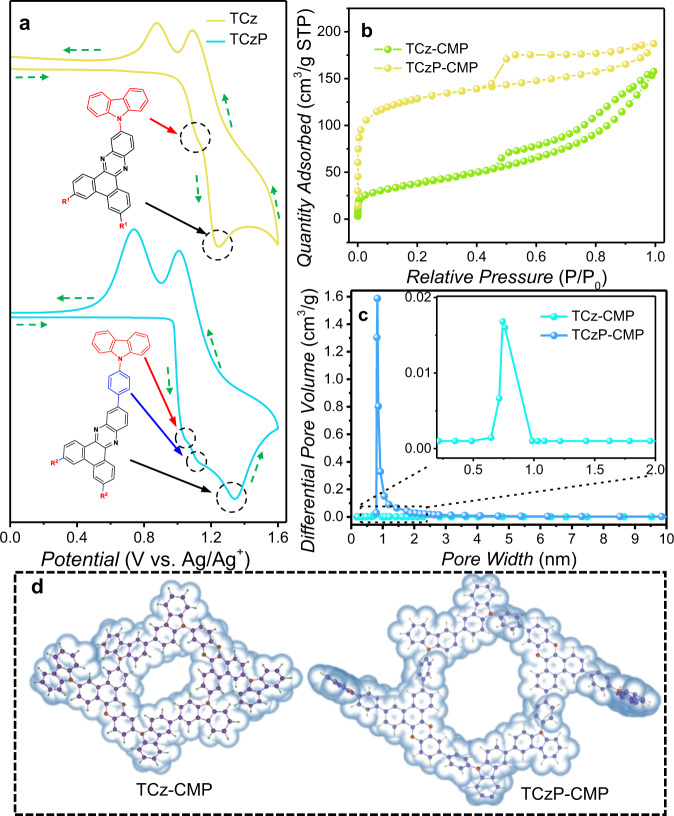
The preparation of CMP films and pore size analysis. **a** The single-cycle CV curve of TCz and TCzP with the scanning potential of 0–1.6V. **b** Nitrogen adsorption/desorption curve of TCz-CMP and TCzP-CMP. **c** Pore size distribution curves of TCz-CMP and TCzP-CMP; the pore size distribution was calculated from the corresponding N_2_ adsorption isotherm using the Horvath-Kawazoe method. **d** The optimal configuration of TCz (left) and TCzP (right) calculated by TDDFT.

In order to further improve the detection performance and the optical stability of these films, the CMP films were prepared by cyclic voltammetry (CV) in a standard three-electrode electrochemical cell^35^. In the single-cycle CV curve, the initial oxidation potentials of TCz and TCzP are observed at 1.03 V and 0.98 V, which are attributed to the oxidation of carbazole group. As the scanning potential increases to 1.11 V and 1.03 V in TCz and TCzP, respectively, indicating more carbazole groups were oxidized and produce more carbazole radical cations (Fig. 3a). Then, an oxidation potential appears at 1.12 V for TCzP, which is assigned to the oxidation of the benzene ring connected to carbazole. As the scanning potential continues to increase, TCz and TCzP generate a peak potential at 1.23 V and 1.35 V, respectively, indicating that DPPZ was oxidized. During the negative scan, two obvious reduction peaks of TCz are observed at 0.88 V and 1.09 V, which correspond to the reduction of dimeric carbazole cations and DPPZ. Correspondingly, the reduction peaks of TCzP are located at 0.74 V and 1.01 V. Accordingly, the high oxidation potentials of 1.2 V and 1.1 V were selected for the preparation of TCz-CMP and TCzP-CMP films, respectively (Supplementary Fig. 20). This oxidation potential can ensure the effective coupling reaction of the carbazole groups while avoiding the oxidation of DPPZ to increase the fluorescence intensity of the CMP films. In this case, the oxidation of carbazoles forms carbazole cation radicals which can quickly undergo a coupling reaction with a neutral carbazole to form a dimeric carbazole cation with a lower oxidation potential, and CMP films are obtained by the redox reaction of the dimeric carbazole cations. In the multicycle CV curves of TCz and TCzP, the increase of the reduction peak indicates that the CMP films are starting to be deposited on the electrode, which is the proof of the formation of CMP films. The CMP films with uniform surface morphology can be observed by high-resolution transmission electron microscope (HRTEM) (Supplementary Fig. 21). Moreover, the film thickness of TCz-CMP and TCzP-CMP can be precisely controlled by scanning cycles. It can be seen that for TCz-CMP and TCzP-CMP films, the film thickness has a good linear relationship with the scanning cycles and increases by 1.05 nm and 2.95 nm per scanning cycle, respectively (Supplementary Fig. 22). The formation of TCz-CMP and TCzP-CMP films was further confirmed by Fourier transform infrared (FT-IR) spectroscopy (Supplementary Fig. 23). TCz-CMP and TCzP-CMP showed a new peak appearing about 804 nm that is assigned to dimeric carbazole, whereas the absorption peaks of DPPZ at 766 nm and 775 nm were almost unchanged before and after EP, indicating that the oxidation of carbazole did not affect DPPZ. In addition, the powder X-ray diffraction (XRD) patterns of TCz-CMP and TCzP-CMP demonstrate a broad and dispersion peak within the 2θ range of 5–40° (Supplementary Fig. 24), indicating the amorphous feature of CMP films, which is expected for the preparation of the CMP films.

To further study the microporous structure of TCz-CMP and TCzP-CMP, we conducted nitrogen adsorption/desorption experiments and evaluated their porosity at 77.3 K (Fig. 3b, Supplementary Note 7). Both TCz-CMP and TCzP-CMP show typical iv-type nitrogen adsorption isotherms according to the IUPAC classification. The obtained curve shows that nitrogen uptake increases sharply at low relative pressures (p/p_0_ less than 0.05), which indicates the existence of a microporous structure. Under relative high pressure (0.4–1.0), the hysteresis loop shows the coexistence of micropores and mesopores. The Brunauer-Emmett-Teller (BET) surface area and a total pore volume of TCzP-CMP were calculated to be 407 m^2^/g and 1.29 cm^3^/g. In a comparison, TCz-CMP has a smaller BET surface area (137 m^2^/g) and total pore volume (0.12 cm^3^/g). Correspondingly, the pore size distributions of TCzP-CMP and TCz-CMP are 0.81–1.0 nm and 0.71–0.74 nm, respectively (Fig. 3c). After the optimization by TDDFT, the possible microporous structures of TCzP-CMP and TCz-CMP were obtained (Fig. 3d). However, the pore size of TCz-CMP is smaller than that of DCP (0.84 nm), which is not conducive to the diffusion of DCP into the interior. In contrast, the pore size of TCzP-CMP is suitable for the diffusion of DCP.

### Adsorption performance test of CMP

**Fig. 4:**
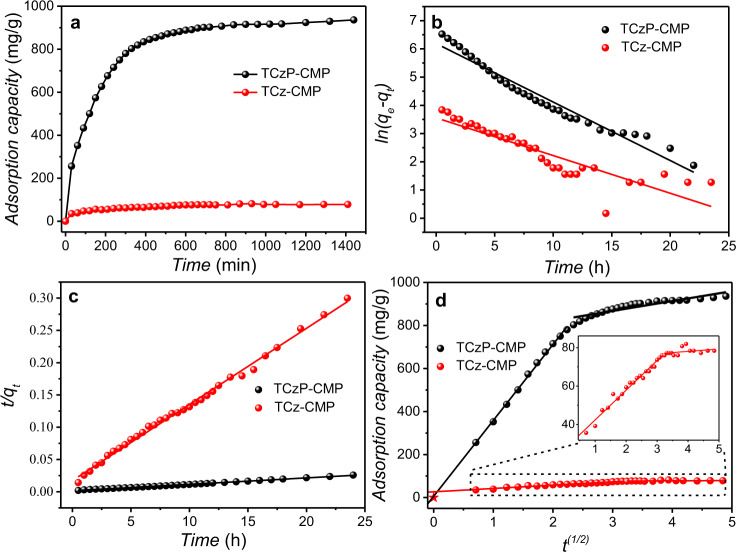
Adsorption performance of CMP to DCP vapors. **a** Adsorption capacity of the TCz-CMP and TCzP-CMP to DCP; **b** PFO model; **c** PSO model; **d** intra-particle diffusion model. The original adsorption data are in Source Data.

The adsorption capacity of CMP to the analytes is an important factor affecting the sensing performance, so a simple self-made adsorption system was first built to evaluate the adsorption capacity of TCz-CMP and TCzP-CMP to DCP vapors. This is a simple and effective gas adsorption method with a detection sensitivity of 0.1 mg. The CMP powders (30 mg) were placed in the sample chamber, and DCP vapor was injected into the chamber through a gas pump, and the gas adsorption capacity of CMP was verified by weighing the change of the chamber mass (Supplementary Figs. 25–27, Supplementary Note 8). It is found that the adsorption capacity of the two CMPs to DCP vapors increases rapidly within 200 min, which is attributed to the existence of sufficient action sites in CMPs (Fig. 4a). Then, with the prolongation of exposure time, the adsorption capacity shows a slow tendency, which is due to the occupation of most sites, resulting in the gradual decrease of adsorption rate, and finally reaches equilibrium at 1400 min. The maximum adsorption capacity of TCzP-CMP can reach 936 mg/g, while TCz-CMP is only 78 mg/g. This may be because TCzP-CMP has a larger pore structure and specific surface area than TCz-CMP, which is more conducive to the diffusion of DCP molecules into CMP. Then, in order to understand the adsorption process in-depth, the adsorption kinetics were evaluated using the pseudo-first-order (PFO) (Eq. 1), pseudo-secondary (PSO) (Eq. 2) model and intra-particle diffusion model (Eq. 3)^36,37^.

Comparing the two correlation coefficients (*R*^2^) of TCz-CMP and TCzP-CMP to DCP vapors, the PSO model (*R*^2^ > 0.99) has a better correlation than the PFO model (*R*^2^ < 0.96), which indicates that the PSO model is more suitable for describing the adsorption process for DCP than the PFO model (Fig. 4b, c). The results show that the adsorption of DCP by TCz-CMP and TCzP-CMP is a chemical adsorption process, which may depend on the nucleophilic substitution reaction and electronic interaction between the two CMPs and DCP^37^. The experimental adsorption capacity of TCz-CMP of 78 mg/g is much lower than that of TCzP-CMP (936 mg/g), once again proving that the pore size directly affects the diffusion of DCP. The calculated equilibrium adsorption capacity *q*_e_ (976 mg/g) of TCzP-CMP is close to the experimental value. Furthermore, the intra-particle diffusion model is adopted to fit experimental data, revealing the rate-controlling steps in the DCP adsorption process. It can be observed that there are two linear relationships in the entire time range of the adsorption process, which indicate that surface adsorption and intra-particle diffusion synergistically affect the adsorption process (Fig. 4d). For TCzP-CMP, the fitting line of intra-particle diffusion almost passes through the origin (C_1_ = 6), which proves that the intra-particle diffusion of TCzP-CMP is a speed-controlled step in the first adsorption stage of DCP, and there may be no boundary effect. Concurrently, we found that TCzP-CMP not only has good adsorption performance for DCP vapors but also for other CWA simulants, such as DCNP, DMMP, TEP, and 2-CEES. Similarly, the adsorption kinetics of PFO, PSO, and intra-particle diffusion models were evaluated (Supplementary Fig. 26). It can be seen that the adsorption of these CWA analogs by TCzP-CMP is more consistent with the PSO model, which may depend on the point-to-dipole π interaction^36,38^ (Supplementary Tables 5, 6). More importantly, it has been proved that the adsorption capacity of these five CWA simulants by TCzP-CMP is 2–3 times that of activated carbon (Supplementary Table. 7). Therefore, this TCzP-CMP as the adsorption material for CWA has potential application in military protection.

### Optical properties of CMP films

Compared with the spin-coated films of TCz and TCzP monomers, the emission peaks of TCz-CMP and TCzP-CMP films are red-shifted by 60 nm and 41 nm, respectively, which are ascribed to the extended π-conjugate structure (Supplementary Fig. 28). Meanwhile, the absorption peaks of TCz-CMP and TCzP-CMP films due to extended π-conjugate structure become wide and shift to long wavelengths (Supplementary Fig. 29). To study the fluorescence stability of these two CMP films, the TCz-CMP films and TCzP-CMP films were continuously irradiated under the excitation light with the maximum excitation wavelength at 450 nm and 440 nm for 90 s. The fluorescence intensity of the TCz-CMP films nearly has no changes, while that of the TCzP-CMP films is only quenched by 7%. Both the TCz-CMP and TCzP-CMP films demonstrate much better stability than their monomers films because of their cross-linked structure, thus effectively overcoming the photobleaching problems, which is fully for practical applications (Supplementary Fig. 30).

### Sensing performance of CMP films

**Fig. 5:**
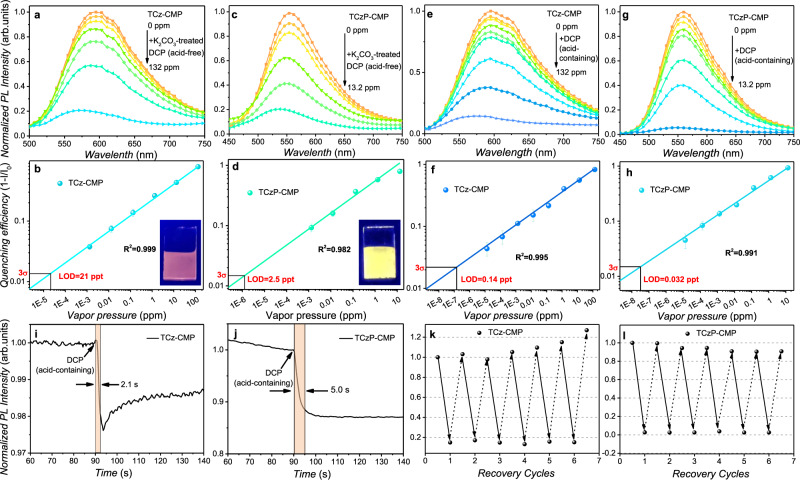
Sensing performance of CMP films. **a**, **c** The fluorescence intensity of TCz-CMP and TCzP-CMP films to K_2_CO_3_-treated DCP vapors (acid-free). **b**, **d** The quenching efficiency of TCz-CMP and TCzP-CMP films exposed to K_2_CO_3_-treated DCP vapors (acid-free); the inset is a photo of CMP films excited at 365 nm. **e**, **g** The fluorescence intensity of TCz-CMP and TCzP-CMP films to acid-containing DCP vapors. **f**, **h** The quenching efficiency of TCz-CMP and TCzP-CMP films exposed to acid-containing DCP vapors. **i**, **j** Response time of TCz-CMP and TCzP-CMP films to acid-containing DCP vapors. **k**, **l** The recovery test of TCz-CMP and TCzP-CMP films to acid-containing DCP vapors (Solid arrow: the quenched process of CMP films by 50 mL 30 ppm DCP vapors; dash arrow: the recovery process of CMP films by injecting 50 mL 10% ammonia). Among them, the thickness of TCz-CMP and TCzP-CMP films are 45 nm and 250 nm, respectively. Error bars stand for standard deviation (*n* = 3).

First, the fluorescence response of CMPs films to acid-free DCP vapors was performed (Fig. 5a, c). As a comparison, the LOD of TCzP-CMP (2.5 ppt) to acid-free DCP vapors is an order of magnitude lower than that of TCz-CMP (21 ppt) (Fig. 5b, d). The sensing performance can be attributed to the susceptible on-off effect of hybridization and de-hybridization of HLCT materials, and their microporous and extended conjugate structures. To verify that the CMP films with HLCT state properties have higher sensitivity to DCP than other fluorescent materials, the fluorescence responses of these two CMP films to DCP vapors (acid-containing) were investigated in air atmosphere (Fig. 5e, g). Both TCz-CMP and TCzP-CMP films are more sensitive to acid-containing DCP vapors than acid-free DCP, and their LODs were 0.032 ppt and 0.14 ppt, respectively. This is attributed to the fact that CMP films are more sensitive to HCl than DCP, and the smaller HCl diffuse into the films is more easily (Fig. 5f, h; Supplementary Fig. 31). More importantly, TCz-CMP and TCzP-CMP films have a rapid response to the low concentration of DCP vapors (acid-containing) within 2.1 s and 5 s (Fig. 5i, j), which fully meets the requirements of real-time detection. Furthermore, it is found that the fluorescence intensity of the two CMP films quenched by high-concentration DCP vapors (30 ppm) can be restored to the original level after injecting 50 mL 10% ammonia gas (Fig. 5k, l). Even though recycled for six times, the two CMP films still showed a good response to DCP vapors (acid-containing). Further, the fluorescence responses of the two CMP films to the possible interferences were investigated. Upon exposure to DCP (acid-free, 1.32 ppm), ethanol (780 ppm), TEP (99 ppm), water (32000 ppm), trifluoroacetic acid (TFA 13 ppm), DCNP (2 ppm), DMMP (200 ppm), triethylamine (1090 ppm), 2-CEES (38 ppm), pyridine (25 ppm), acetone (816 ppm), and n-hexane (21 ppm) vapors, respectively, the TCz-CMP and TCzP-CMP films exhibit much higher selectivity to DCP (Supplementary Fig. 32). It should be noted that TCz-CMP film is more sensitive to HCl (1 ppm) than to acid-containing DCP vapors (1.32 ppm) because of its smaller pore size, which is more conducive to the penetration of HCl molecules.

### System assembly and testing

**Fig. 6:**
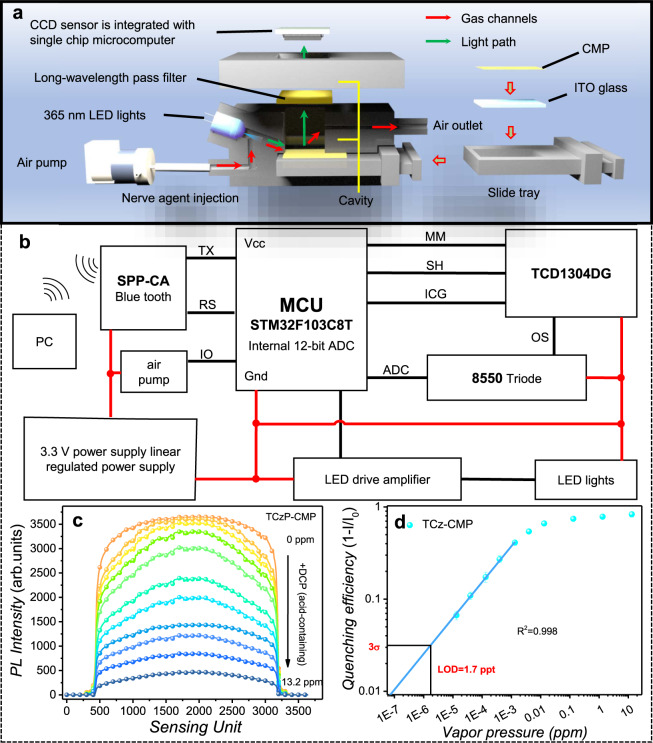
Device design and testing. **a** The internal structure diagram of the portable detection system. **b** Schematic diagram of the circuit of the detection system. **c** Fluorescence signal data output by the portable detection system, the abscissa is the sensing unit numbers, and the ordinate is the corresponding fluorescence intensity. **d** The quenching efficiency of TCzP-CMP films to DCP vapors (acid-containing) in the portable detection system (the data output are obtained after the integral area is normalized by the instrument), 3σ is three times standard deviation. Among them, the thickness of TCzP-CMP films is 250 nm.

To verify the practical applicability of CMP films, a portable fluorescent detection system consisting of intake pump, TCzP-CMP film, LED (365 nm), optical recognition detection system, Bluetooth transceiver, and an optimized sealed cavity was built (Fig. 6a). The working principle of the device is as follows: using STM32 as the microcontroller unit (MCU), controlling the switch of the air pump and driving the linear charge-coupled device (CCD) sensor to collect signals, and then send the signals to the upper computer (PC) through the Bluetooth transceiver (Fig. 6b). The switch of the LED light is synchronized with the CCD signal collection. Then, the various level of DCP vapors were injected into the sealed cavity for 10 seconds through the intake pump and the fluorescence signal was analyzed by the operating software. It can be observed that the changes of fluorescence intensity to DCP vapors (acid-containing) show a good linearity (*R*^2^ = 0.998), and the LOD can be determined as 1.7 ppt (Fig. 6c, d). In addition, the portable fluorescence detection systems with a Bluetooth device can realize remote and wireless monitoring the DCP vapors, thereby avoiding the risk of direct detection by personnel. The fluorescent system equipped with TCzP-CMP films shows rapid and sensitive performances for DCP detection, indicating that they can meet the requirements of practical applications.

## Discussion

In conclusions, two fluorescent molecules (TCz and TCzP) with HLCT excited state were synthesized, and showed high sensitivity to DCP vapors. In order to overcome the photobleaching problem of these two molecules and explore the influence of microstructure on sensing, two CMP films (TCz-CMP, TCzP-CMP) were prepared using TCz and TCzP as precursor molecules through a simple and effective EP method. More importantly, the thickness of CMP films has a good linear relationship with the scanning cycles, and the thickness increases by 1.05 nm (TCz-CMP) and 2.95 nm (TCzP-CMP) in each scanning cycle. Moreover, TCzP-CMP has a larger BET surface area (407 m^2^/g) and total pore volume (1.29 cm^3^/g) than that of TCz-CMP. Correspondingly, the pore size distributions of TCzP-CMP and TCz-CMP are 0.81–1.0 nm and 0.71–0.74 nm, respectively. Taking the advantages of CMP films, such as the inherent microporous structure, the extended conjugated system, and the highly susceptible on-off effect of the HLCT excited state, TCz-CMP and TCzP-CMP are highly sensitive to DCP, and the LOD can reach 21 ppt and 2.5 ppt. This strategy provides a new idea for the future development of gas sensors. Accordingly, the TCzP-CMP films are implanted in a self-built portable detection system, which realizes remote and wireless detection of DCP with the LOD of 1.7 ppt and verifies the practical application of the TCzP-CMP films. In addition, the equilibrium adsorption capacity of TCzP-CMP can reach 936 mg/g, which is ~3 times that of activated carbon. Therefore, TCzP-CMP is a promising dual-functional material for DCP sensing and military protection.

## Methods

### Chemicals and measurement

All the reagents were obtained from Aldrich, Kanto Chemicals, and TCL unless otherwise specified. DCP purchased from sigma Aldrich. Unless otherwise specified, all reagents are used directly after purchase. Dry toluene is obtained by adding metallic sodium to toluene and refluxing for 8 h. All chromatographic separations were performed on silica gel (200–300 mesh). The ^1^H NMR and ^31^P NMR were recorded on a Bruker AVANCE III HD spectrometer at 500 MHz, using CDCl_3_ as the solvent at 298 K. TMS was used for ^1^H NMR and phosphoric acid was used for ^31^P NMR spectra as internal standards. The MALDI-TOF-MS mass spectra are recorded using an AXIMA-CFRTM plus instrument. UV-vis absorption spectra are recorded on an Agilent Cary100 spectrophotometer. Fluorescent spectra are measured with a Shimadzu RF-6000. The absolute quantum efficiency is measured by HORIBA QM8000 spectrometer. Lifetimes are measured with a FLS-980 on an EPL-375 optical laser. The CMP film thickness is recorded on Bruker Countor GT K &, KLA-Tencor D120 step profiler. FT-IR spectra were recorded on a PerkinElmer Spectrum 400 infrared spectrometer. XRD patterns (PANalytical B.V. X, Pert3 Powder diffractometer). The morphology of the CMP film was photographed on a transmission electron microscope (JEOL JEM-2100).

### Synthesis of compounds TCz

A mixture of 3,6,11-tribromodibenzo[a,c]phenazine 517 mg (1 mmol), carbazole 1003 mg (6 mmol), CuI 38 mg (0.2 mmol), trans-1,2-Diaminocyclohexane 980 µL (8 mmol), K_3_PO_4_ 955 mg (4.5 mmol), and dry toluene 100 mL was added into a 250 mL flask. Vacuum to remove air under liquid nitrogen freezing, and then reflux for 48 h under nitrogen protection. After cooling to room temperature, it was extracted with dichloromethane/water, and the organic layer was dried with MgSO_4_. The mixture was purified through a silica gel column, using dichloromethane/petroleum ether (v/v = 1:4) as the eluent, the crude product obtained was recrystallized with toluene/anhydrous methanol, and orange-yellow powder (316 mg) was obtained with a yield of 41%. ^1^H NMR (500 MHz, CDCl_3_) 9.75 (2 H, dd, J 18.7, 8.6), 8.74 (2 H, s), 8.68–8.60 (2 H, m), 8.29–8.13 (7 H, m), 8.07 (2 H, dd, J 13.8, 8.5), 7.74 (2 H, d, J 8.0), 7.61 (4 H, t, J 7.2), 7.52 (2 H, t, J 7.7), 7.43 (6 H, dt, J 15.7, 7.3), 7.33 (4 H, dd, J 12.8, 7.0). ^13^C NMR (151 MHz, CDCl_3_) δ 143.08, 142.63, 142.17, 141.35, 140.59, 140.34, 140.21, 139.40, 133.24, 133.06, 131.24, 129.44, 129.34, 129.19, 128.73, 128.57, 127.11, 126.33, 125.68, 124.05, 123.83, 121.01, 120.86, 120.56, 109.93, 109.67. MALDI-TOF-MS (m/z): [M]^+^ calcd. for C_56_H_33_N_5_, 776.131.

### The synthesis of compound TCzP

K_2_CO_3_ 2239 mg (16.2 mmol), water 54 mL, ethanol 35 mL, and toluene 100 mL were added to a 500 mL flask. Next, the mixture of 3,6,11-tribromodibenzo[a,c]phenazine 517 mg (1 mmol), (4-(9H-carbazol-9-yl)phenyl) boronic acid 1291 mg (4.5 mmol)), Pd (PPh_3_)_4_ 173 mg (0.15 mmol) was added, and applying vacuum to remove air under the liquid nitrogen freezing, and then react at 85 °C for 48 hunder nitrogen protection. After cooling to room temperature, it was extracted with Toluene/water, and the organic layer was dried with MgSO_4_. The mixture was purified by silica gel column using dichloromethane/petroleum ether (v/v = 1:1.5) as the eluent to obtain the crude product. Toluene/anhydrous methanol was recrystallized to obtain yellow powder with a yield of 53% (532 mg). ^1^H NMR (500 MHz, CDCl_3_) 9.66–9.55 (2 H, m), 8.98 (2 H, s), 8.70 (1 H, d, J 1.9), 8.50 (1 H, d, J 8.7), 8.27 (1 H, dd, J 8.8, 2.0), 8.19 (6 H, t, J 6.9), 8.16–8.13 (2 H, m), 8.13–8.08 (6 H, m), 7.83–7.76 (6 H, m), 7.59–7.52 (6 H, m), 7.50–7.44 (6 H, m), 7.34 (6 H, dd, J 15.4, 8.1). ^13^C NMR (151 MHz, CDCl_3_) δ 143.08, 142.63, 142.17, 141.35, 140.59, 140.34, 140.21, 139.40, 133.24, 133.06, 131.24, 129.44, 129.34, 129.19, 128.73, 128.57, 127.11, 126.33, 125.68, 124.05, 123.83, 121.01, 120.86, 120.56, 109.93, 109.67. MALDI-TOF-MS (m/z): [M]^+^ calcd. for C_74_H_45_N_5_, 1004.527.

### Preparation of CMP films

CMP films were prepared using a CH Instruments CHI660E electrochemical analyzer in a three-electrode cell. The reference electrode, working electrode and counter electrode correspond to Ag/Ag^+^ non-aqueous electrode (0.1 M), indium tin oxide (ITO: 1.0 × 1.5 cm) and titanium plate titanium sheet (1.0 × 1.5 cm). Then, the electrolyte solution was added in cell. The electrolyte solution is a mixture of precursor molecule (TCz or TCzP), tetrabutylammonium hexafluorophosphate (0.1 M), and the dry dichloromethane (5 mL). Here, the concentrations of TCz and TCzP are 5 × 10^−5^ M and 4 × 10^−4^ M, respectively, and the multicycle cyclic voltammetry were adopted for the preparation. The preparation conditions for TCz-CMPs films: scanning cycles: 50 cycles; scanning rate: 100 mV/s; scanning potentials: −0.6–1.2 V. And the preparation conditions for TCzP-CMPs films: scanning cycles: 100 cycles; scanning rate: 200 mV/s; scanning potentials: 0–1.1 V.

The bulky samples were obtained by chemical oxidation with ferrous (III) chloride^28^. The fluorescent material (TCz or TCzP) monomer (0.15 mmol) was dissolved in the chloroform solution (50 mL), and then added dropwise to the chloroform suspension (15 mL) containing ferric chloride (3.6 mmol). After stirring for 48 h at room temperature under nitrogen, methanol (100 mL) was added to the reaction mixture and stirred for another 1 h. The insoluble solid was filtered and vigorously stirred in hydrochloric acid solution (37%, 200 mL) for 2 h. Then the suspension was filtered and washed with water, ammonia and methanol. Finally, the insoluble solid was purified by methanol using a Soxhlet extractor for 24 h, and the resulting powder yields were 53% (TCz-CMP) and 79% (TCzP-CMP).

### Adsorption kinetics formula

The pseudo first-order (PFO) (Eq. 1), PSO (Eq. 2) model, and intra-particle diffusion model (Eq. 3) is as follows^37^:

1 $$\begin{array}{c}{{{{{\rm{ln}}}}}}\left({q}_{e}-{q}_{t}\right)={{{{{\rm{ln}}}}}}{q}_{e}-{k}_{1}t\end{array}$$

2 $$\frac{1}{{q}_{t}}=\frac{1}{{k}_{2}{q}_{e}^{2}}+\frac{t}{{q}_{e}}$$

3 $$\begin{array}{c}{q}_{t}={k}_{i}{t}^{1/2}+C\end{array}$$

where *t* is the exposure time (h), *q*_t_ (mg/g) and *q*_e_ (mg/g) represent the adsorption capacity of DCP at time *t* and equilibrium, respectively. *k*_1_ (1/h), *k*_2_ (g/(mg h)), *k*_i_ (mg/(g h^1/2^)) refer to the rate constant, and *C* (mg/g) is the intercept, which reflects the boundary layer effect.

## Supplementary information

Supplementary Information

## Data Availability

The theoretical calculation results, NMR and MS data (Fig. 2, Supplementary Figs. 2–7, 11, 12, 14–16) used in this study are available in the Harvard Dataverse database under accession code 10.7910/DVN/KZUCKY. Source data are provided with this paper.

## Source data

Source Data
