# Supplementary material for: Controllable synthesis of conjugated microporous polymer films for ultrasensitive detection of chemical warfare agents
Source: Nat Commun. 2022 Sep 3;13:5189. doi: 10.1038/s41467-022-32878-w (PMC9440894; doi:10.1038/s41467-022-32878-w)
Supplement: Supplementary file 1 — Supplementary Information [file 41467_2022_32878_MOESM1_ESM.pdf]

Supplementary Information

Controllable Synthesis of Conjugated Microporous Polymer Films for Ultra-sensitive Detection of Chemical Warfare Agents

Wanqi Mo[1], Zihao Zhu[1], Fanwei Kong[1], Xiaobai Li[1,2*], Yu Chen[1], Huaqian Liu[1], Zhiyong Cheng[1], Hongwei Ma[1,2*] and Bin Li[1,2*]

[1] Key Laboratory of Forest Plant Ecology, Ministry of Education, Engineering Research Center of Forest Bio-Preparation, College of Chemistry, Chemical Engineering and Resource Utilization, Northeast Forestry University, Harbin 150040, P. R. China.

[2] Post-doctoral Mobile Research Station of Forestry Engineering, Northeast Forestry University, Harbin 150040, P. R. China

E-mail: mahw@nefu.edu.cn; lixiaobai2008@126.com; libin82192699@nefu.edu.cn

Supplementary Figures

Supplementary Figure 1. Material Synthesis.

Supplementary Figures 2-3,5-6. NMR spectra.

Supplementary Figures 4,7. MALDI-TOF spectra.

Supplementary Figures 8-10. Sensing performance of polymer precursors.

Supplementary Figures 11-17. Sensing mechanism analysis.

Supplementary Figure 18. Excited state energy.

Supplementary Figure 19. UV-Vis absorption spectra.

Supplementary Figure 20. Electrochemical polymerization.

Supplementary Figure 21. TEM images of CMP films.

Supplementary Figure 22. The optimization of film thickness.

Supplementary Figure 23. FT-IR spectroscopy analysis.

Supplementary Figure 24. XRD pattern of the TCz-CMP and TCzP-CMP.

Supplementary Figure 25. Diagram of CMP adsorption test.

Supplementary Figure 26. CMP adsorption of other toxic agent simulants.

Supplementary Figure 27. The thermogravimetric analysis (TGA) of TCzP-CMP.

Supplementary Figure 28. The fluorescent properties.

Supplementary Figure 29. UV-Vis absorption spectra of spin-coated films and CMP films.

Supplementary Figure 30. Fluorescence intensity of spin-coated films and CMP films based on TCz and TCzP as the function of excitation time in air.

Supplementary Figure 31. Titration experiment of HCl.

Supplementary Figure 32. Interference test.

Supplementary Table

Supplementary Table 1. The photoluminescence efficiency, lifetime, radiation and non-radiation transition rate of compounds in four solvents.

Supplementary Table 2. Solvatochromic UV-PL data for Lippert-Mataga model.

Supplementary Table 3. Summary of the reported LOD of DCP from previous work.

Supplementary Table 4. Summary of the reported LOD of DCP and HCl from this work.

Supplementary Table 5. Kinetic parameters of pseudo-first and pseudo-secondary models of TCz-CMP and TCzP-CMP adsorption of CWA simulants.

Supplementary Table 6. Intra-particle diffusion model parameters of TCz-CMP and TCzP-CMP adsorption of CWA simulants.

Supplementary Table 7. The adsorption capacity of TCzP-CMP and activated carbon on CWA simulants.

Supplementary Notes

Supplementary Note 1. Radiation form calculation.

Supplementary Note 2. Solvatochromic UV-PL data for Lippert-Mataga model.

Supplementary Note 3. Preparation of spin-coated films.

Supplementary Note 4. Fluorescence detection of DCP vapors.

Supplementary Note 6. Reversibility verification of the response mechanism.

Supplementary Note 6. Theoretical Calculations.

Supplementary Note 7. Nitrogen adsorption/desorption measurements.

Supplementary Note 8. Accuracy verification of the adsorption equipment.

Supplementary References

Supplementary Figures

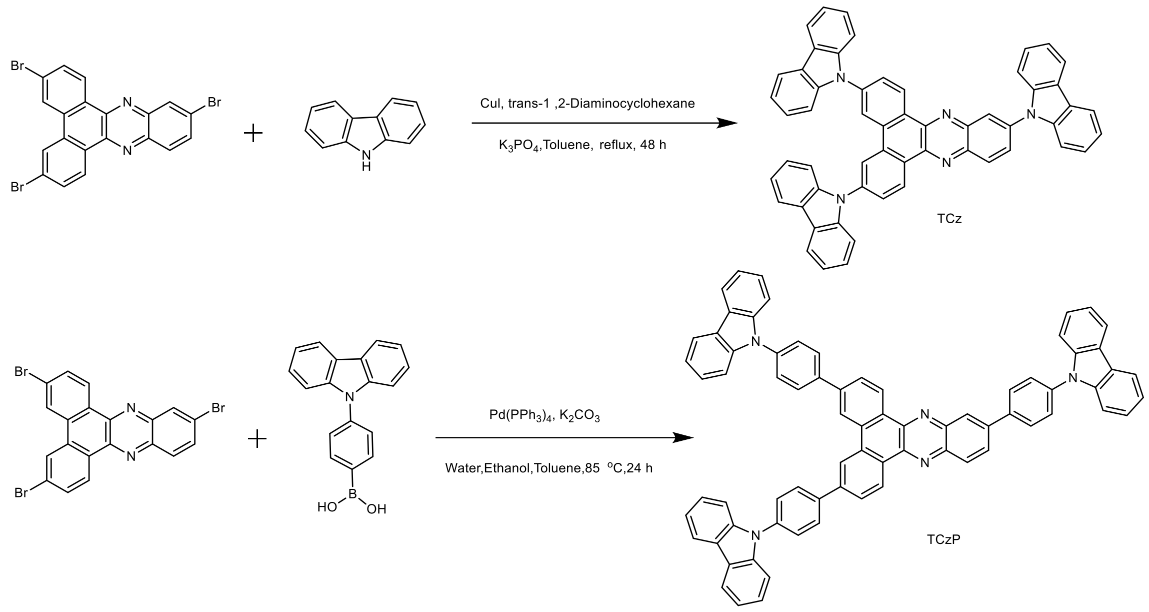

Supplementary Figure 1. Material Synthesis. Synthetic routes of TCz and TCzP based on Ullmann reaction and Suzuki coupling reaction.

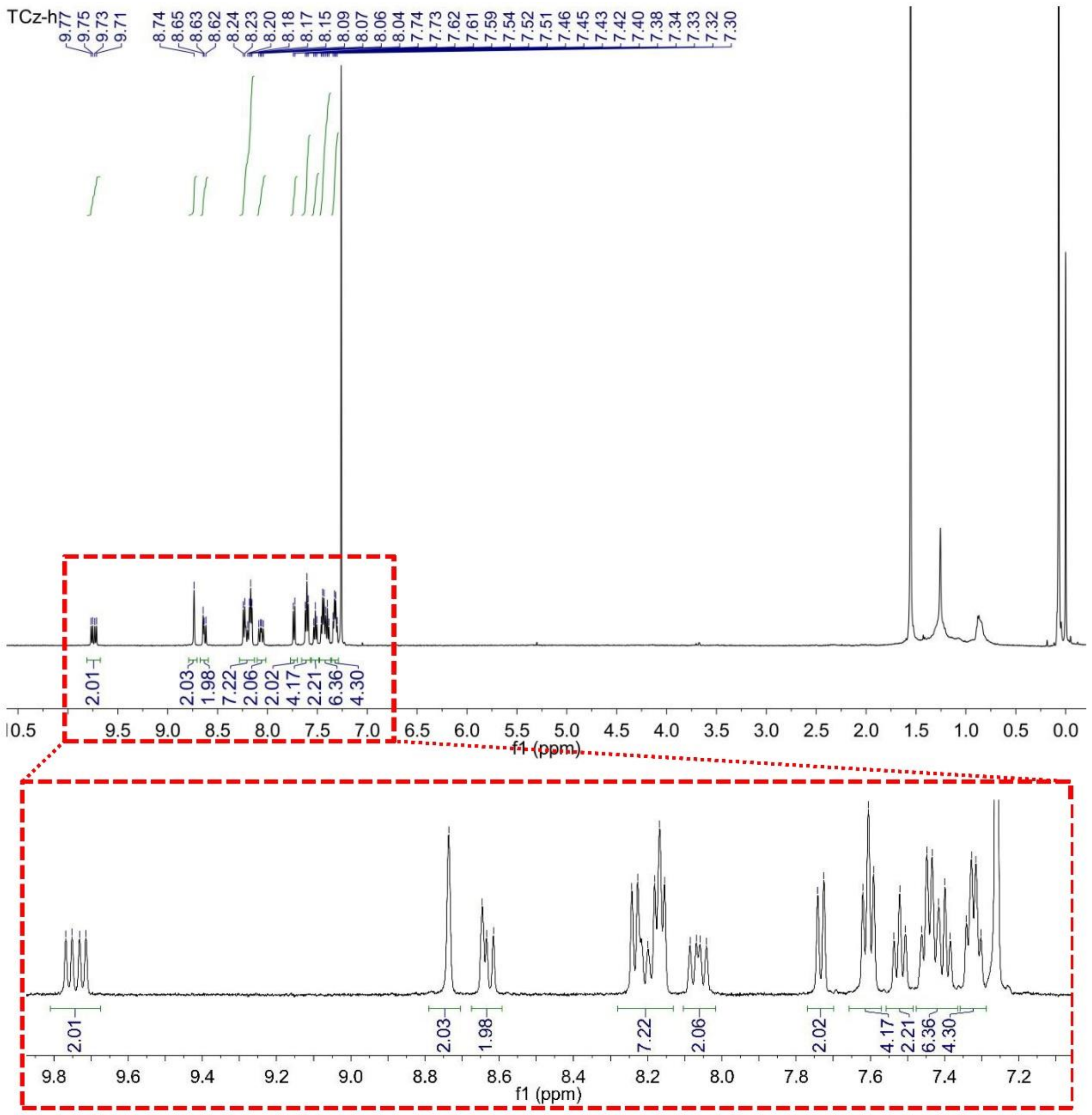

Supplementary Figure 2. ^{1}H NMR spectra of TCz. The peak at 7.26 ppm, 1.56 ppm and 0.5-1.5 ppm correspond to the $CDCl_3$, water, and petroleum ether.

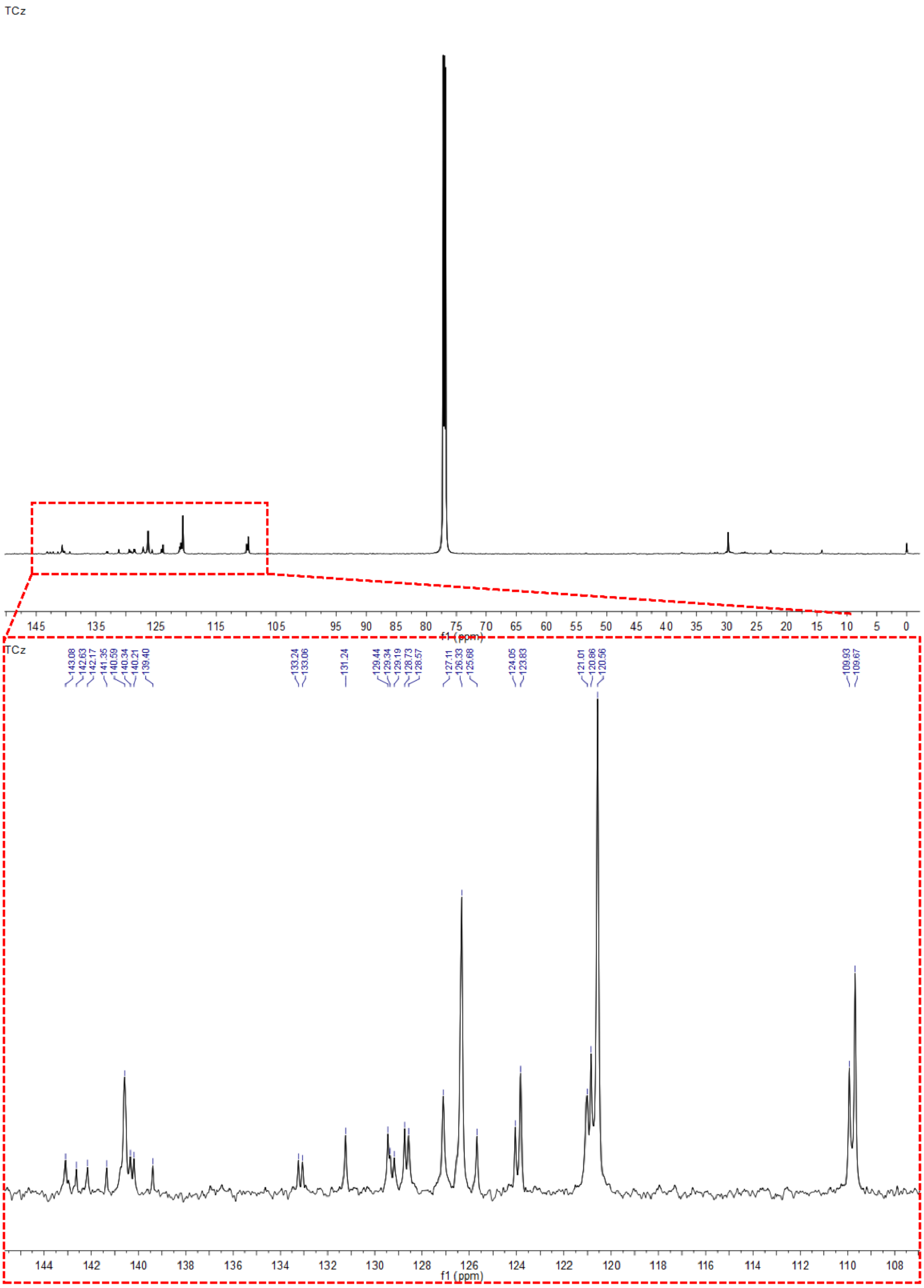

Supplementary Figure 3. ^{13}C NMR spectra of TCz. The peak at 77.16 ppm, 29.71 ppm ,22.66 ppm and 14.12 ppm correspond to the $CDCl_3$ and petroleum ether.

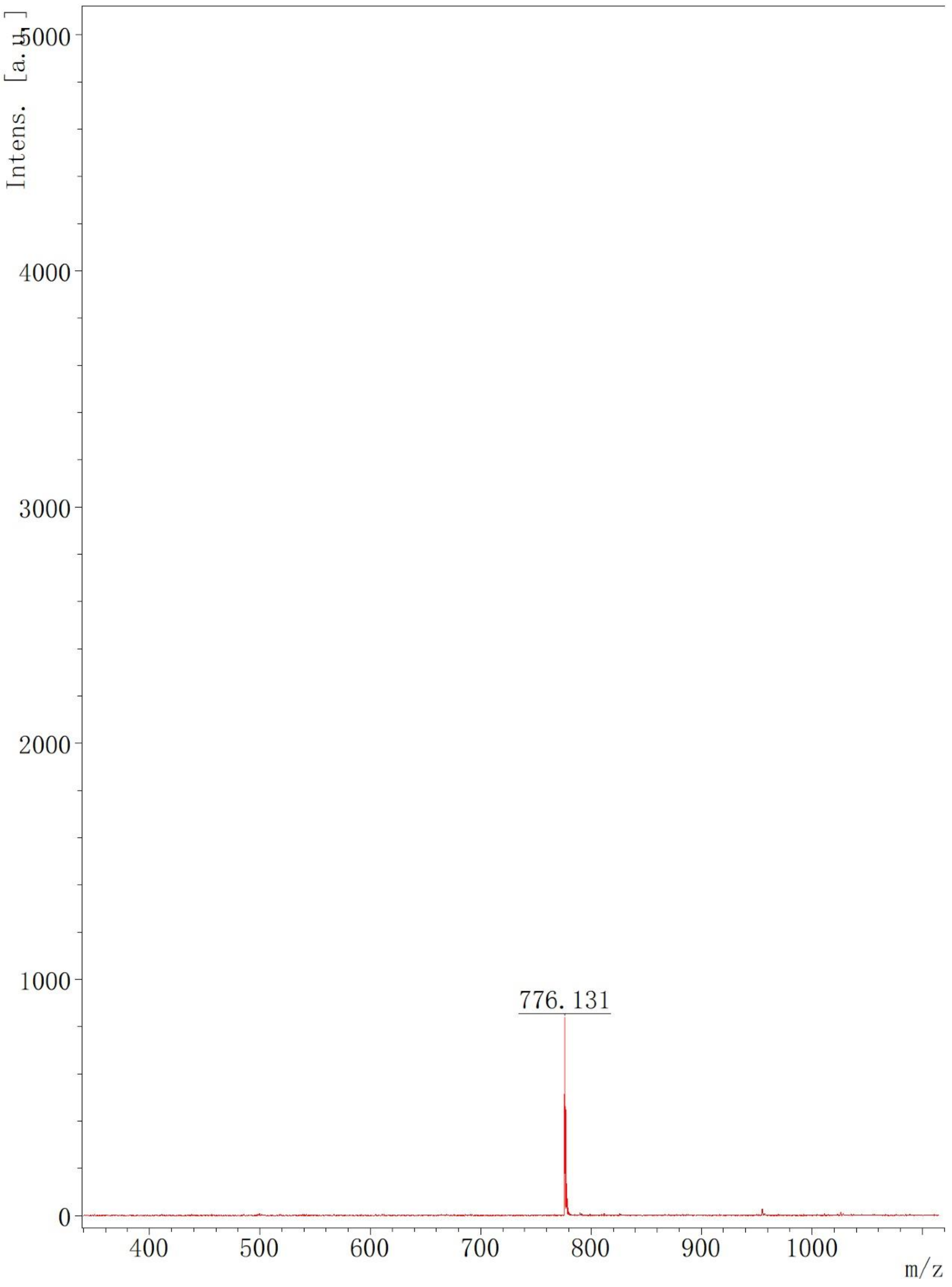

Supplementary Figure 4. MALDI-TOF spectra of TCz.

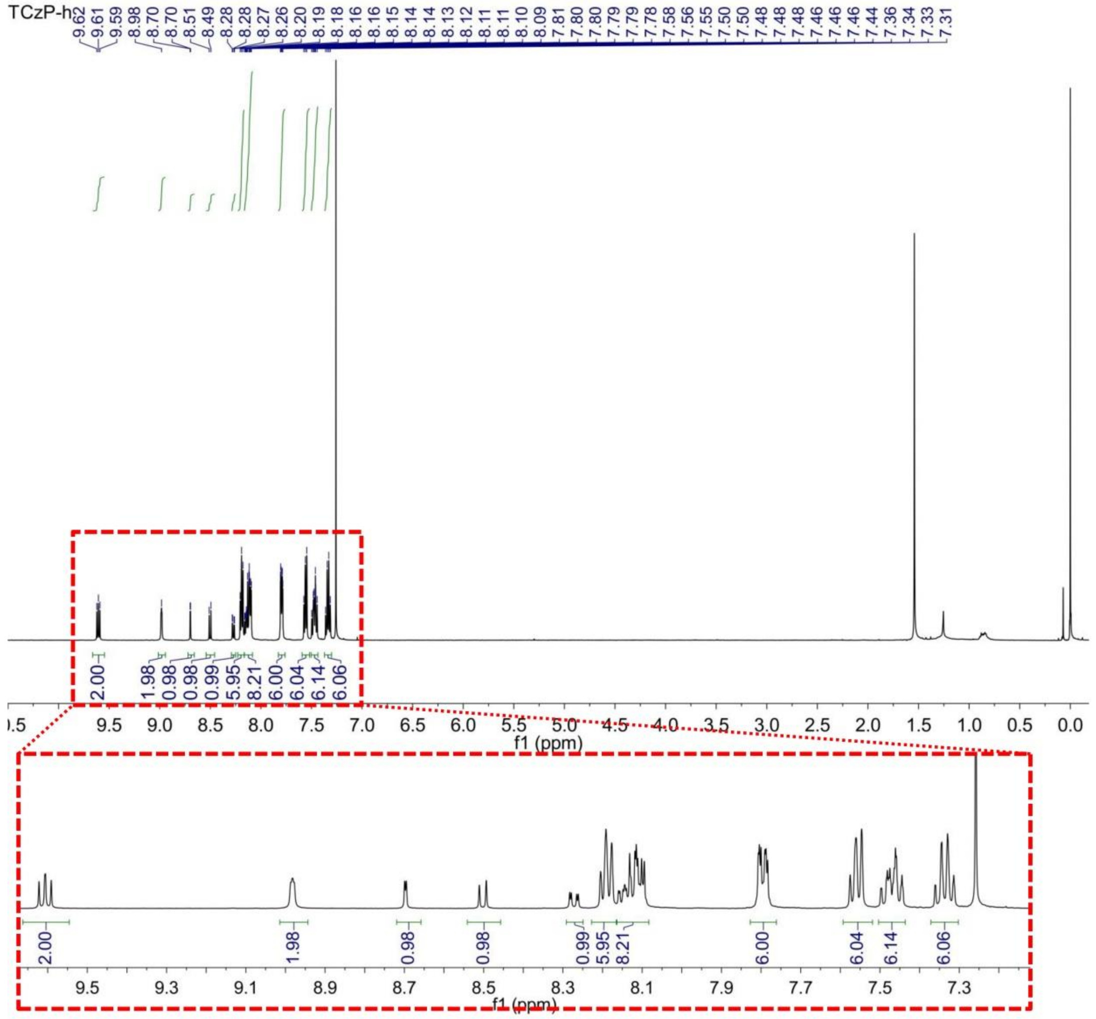

Supplementary Figure 5. ^{1}H NMR spectra of TCzP. The peak at 7.26 ppm, 1.56 ppm and 0.5-1.5 ppm correspond to the $CDCl_3$, water, and petroleum ether.

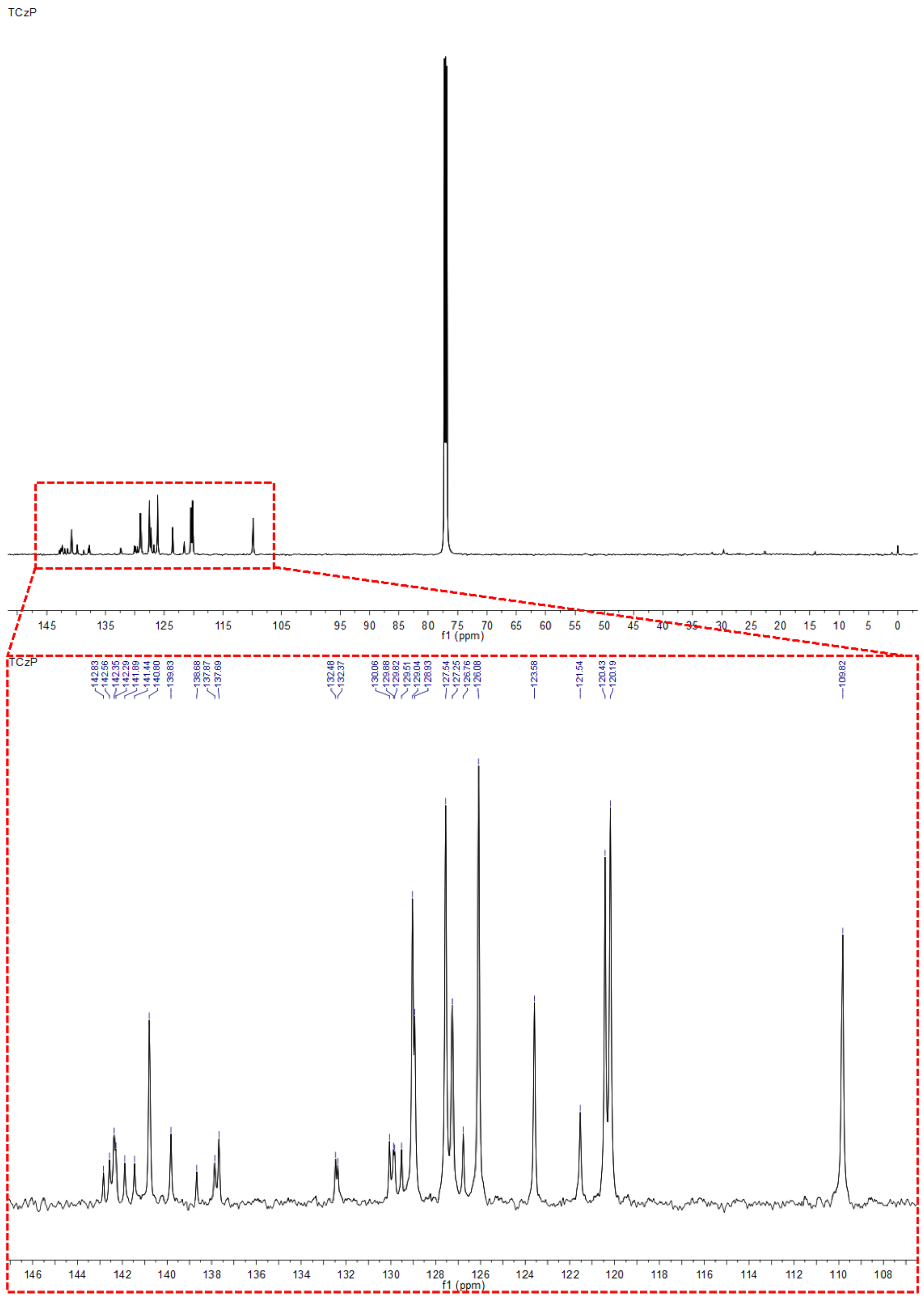

Supplementary Figure 6. ^{13}C NMR spectra of TCzP. The peak at 77.16 ppm, 29.71 ppm ,22.66 ppm and 14.12 ppm correspond to the $CDCl_3$ and petroleum ether.

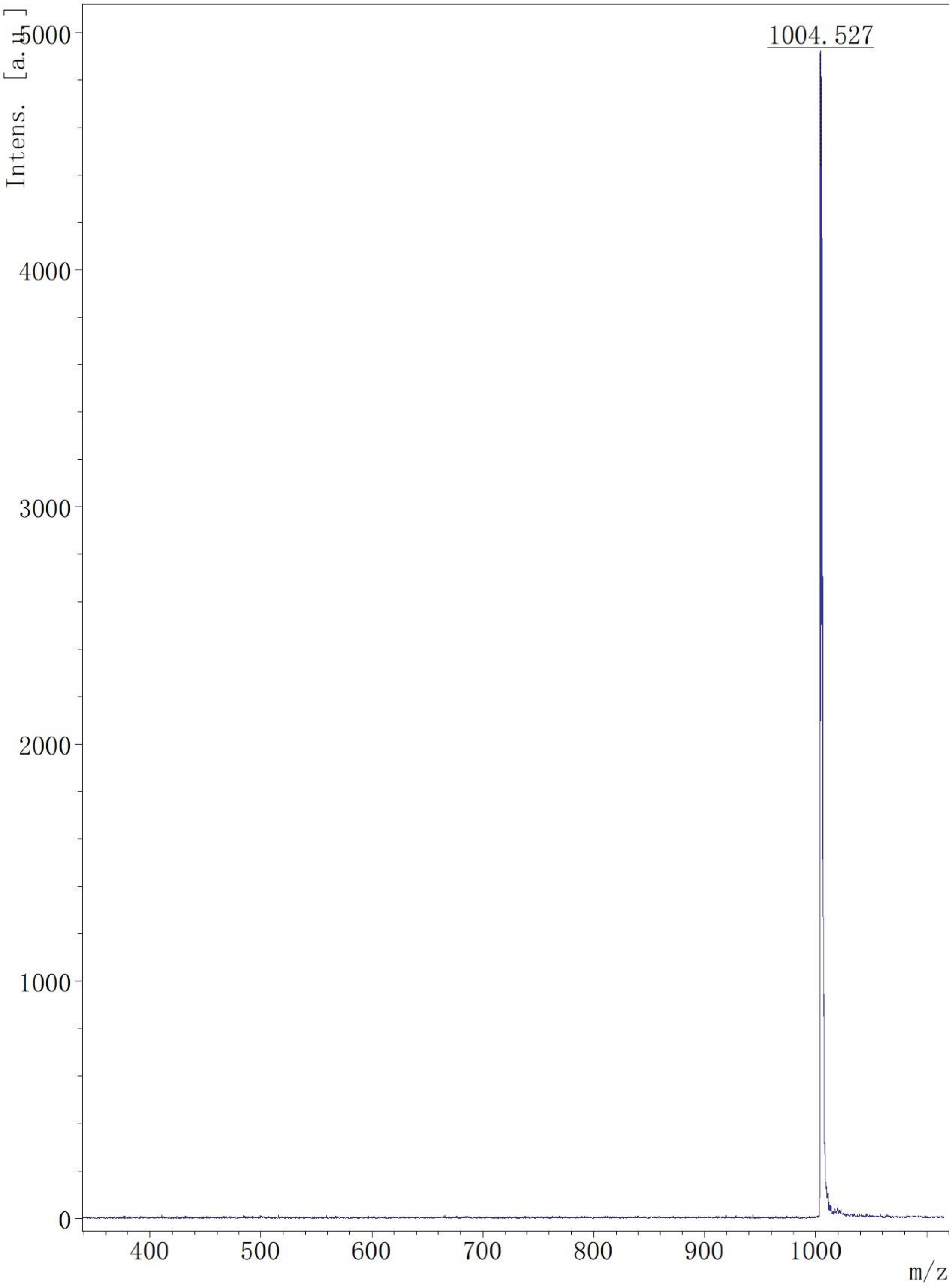

Supplementary Figure 7. MALDI-TOF spectra of TCzP.

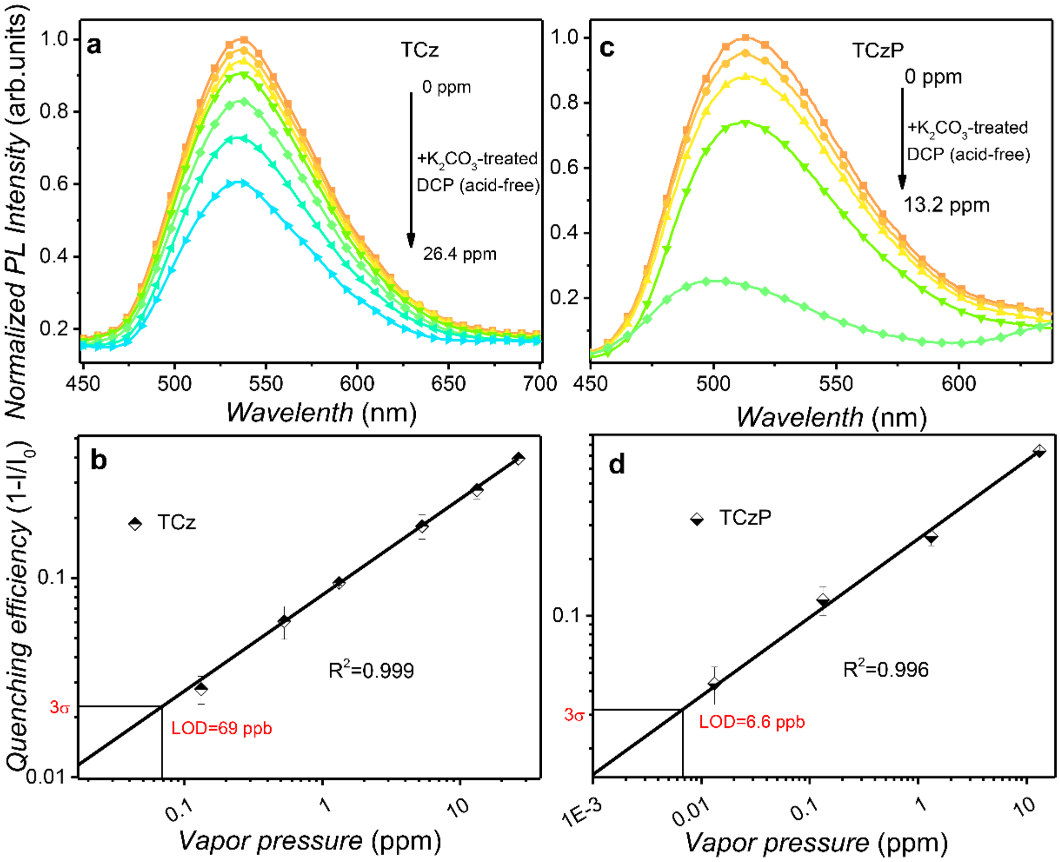

Supplementary Figure 8. The sensing performance of spin-coated film to K_2CO_3-treated DCP vapors (acid-free) under N_2 atmosphere: a the fluorescence intensity of TCz spin-coated films to K_2CO_3-treated DCP vapors (0-26.4 ppm). **b** the quenching efficiency of TCz spin-coated films exposed to K_2CO_3-treated DCP vapors. **c** the fluorescence intensity of TCzP spin-coated films to K_2CO_3-treated DCP vapors (0-13.2 ppm). **d** the quenching efficiency of TCzP spin-coated films exposed to K_2CO_3-treated DCP vapors.

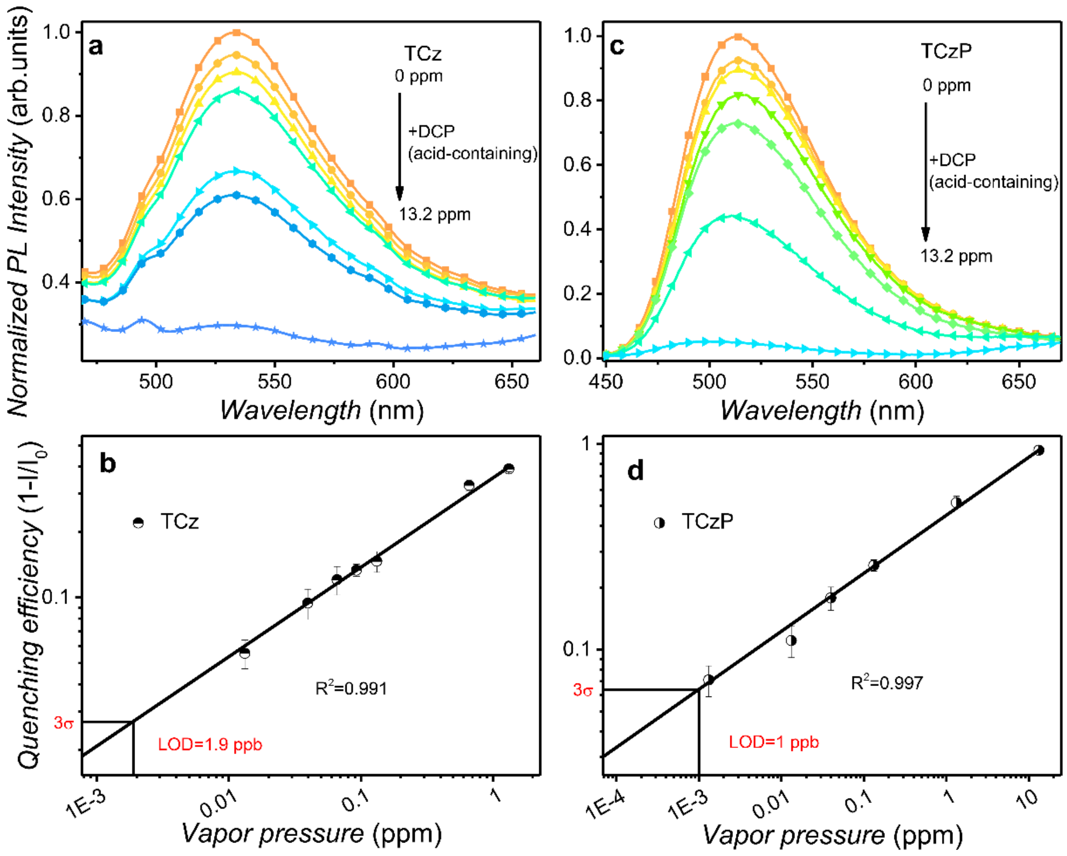

Supplementary Figure 9. Sensing performance of polymer precursors. a The fluorescence intensity of TCz spin-coated films to DCP vapors (0-13.2 ppm). **b** The quenching efficiency of TCz spin-coated films exposed to DCP vapors. **c** The fluorescence intensity of TCzP spin-coated films to DCP vapors (0-13.2 ppm). **d** The quenching efficiency of TCzP spin-coated films exposed to DCP vapors. Among them, the thickness of TCz and TCzP spin-coated films are 4 nm and 6 nm, respectively. Error bars stand for standard deviation (n = 3). The tested DCP vapors were prepared in air and contained acid impurities.

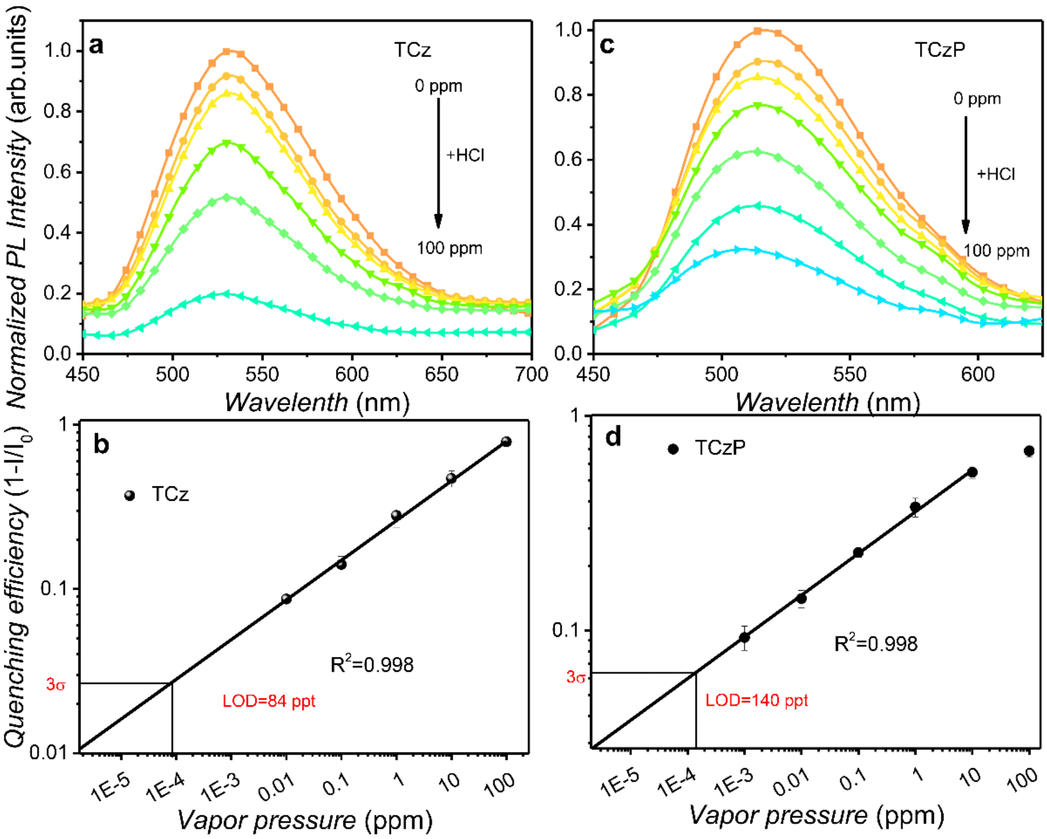

Supplementary Figure 10. **Titration experiment of HCl. a** The fluorescence intensity of TCz spin-coated films to HCl vapors (0-100 ppm). **b** The quenching efficiency of TCz spin-coated films exposed to HCl vapors. **c** The fluorescence intensity of TCzP spin-coated films to HCl vapors (0-100 ppm). **d** The quenching efficiency of TCzP spin-coated films exposed to HCl vapors. Among them, the thickness of TCz-CMP and TCzP-CMP films are 45 nm and 250 nm, respectively. Error bars stand for standard deviation (n = 3).

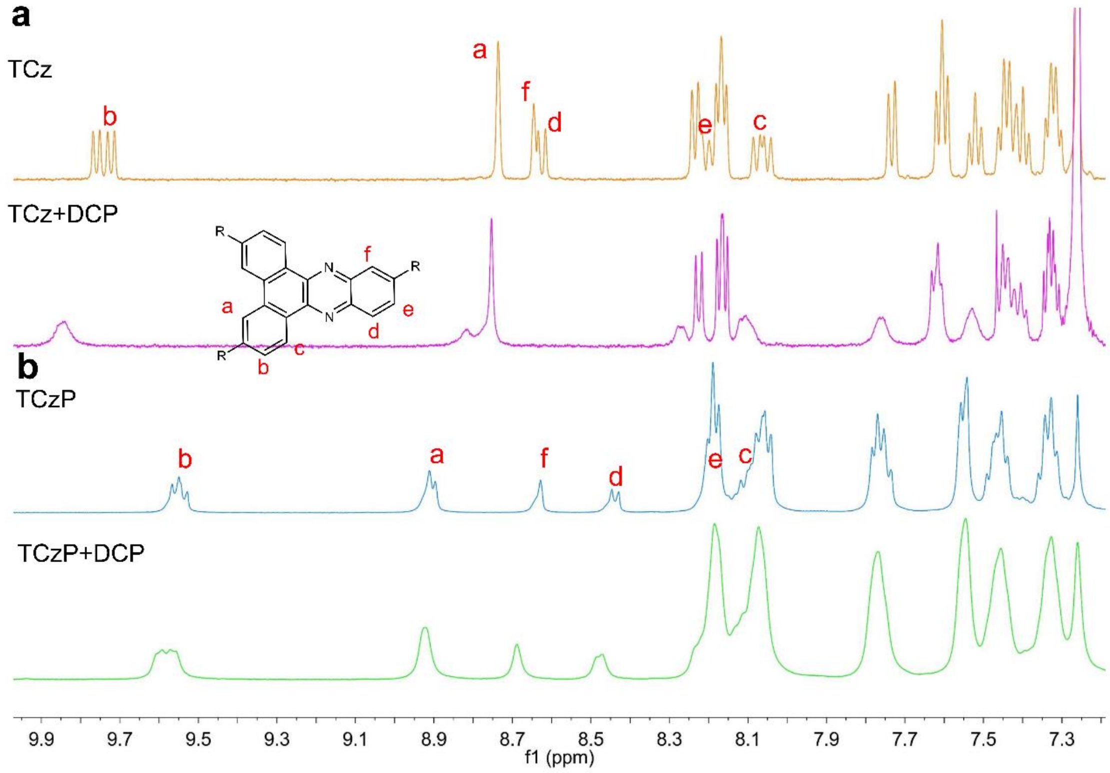

Supplementary Figure 11. ^{1}H NMR mechanism analysis. a ^{1}H NMR titration experiments of TCz before and after the addition of DCP (20 μM). **b** ^{1}H NMR titration experiments of TCzP before and after addition of DCP (20 μM).

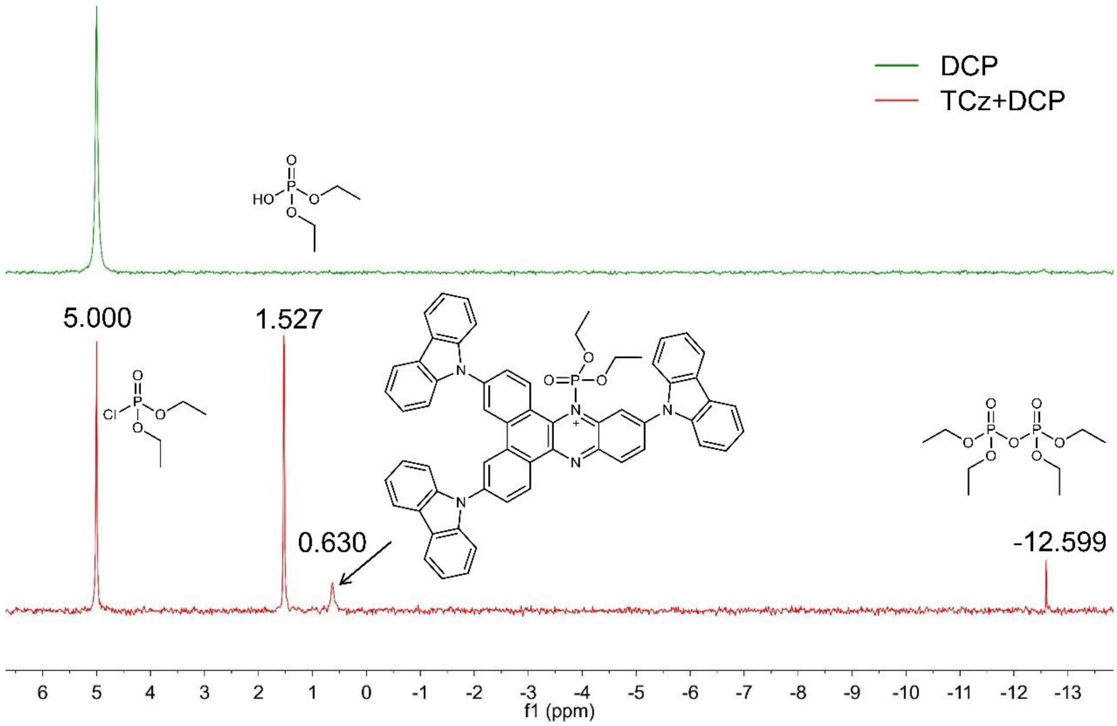

Supplementary Figure 12. ^{31}P NMR mechanism analysis. ^{31}P NMR titration experiments of DCP before and after the addition of TCz.

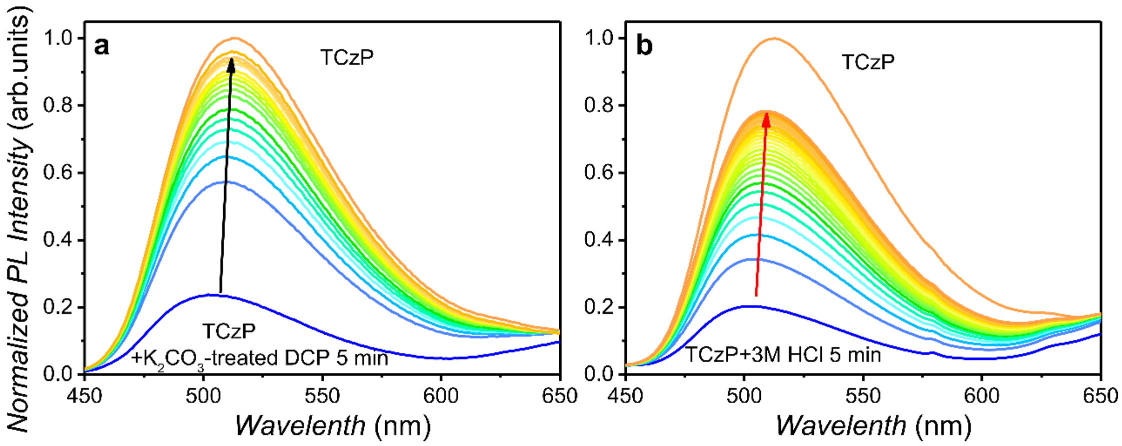

Supplementary Figure 13. The fluorescence recovery spectra of TCzP spin-coated films quenched by (**a**) K_2CO_3-treated DCP vapors and (**b**) HCl vapors produced by 3 M hydrochloric acid under the N_2 blowing rate of 700 mL/min.

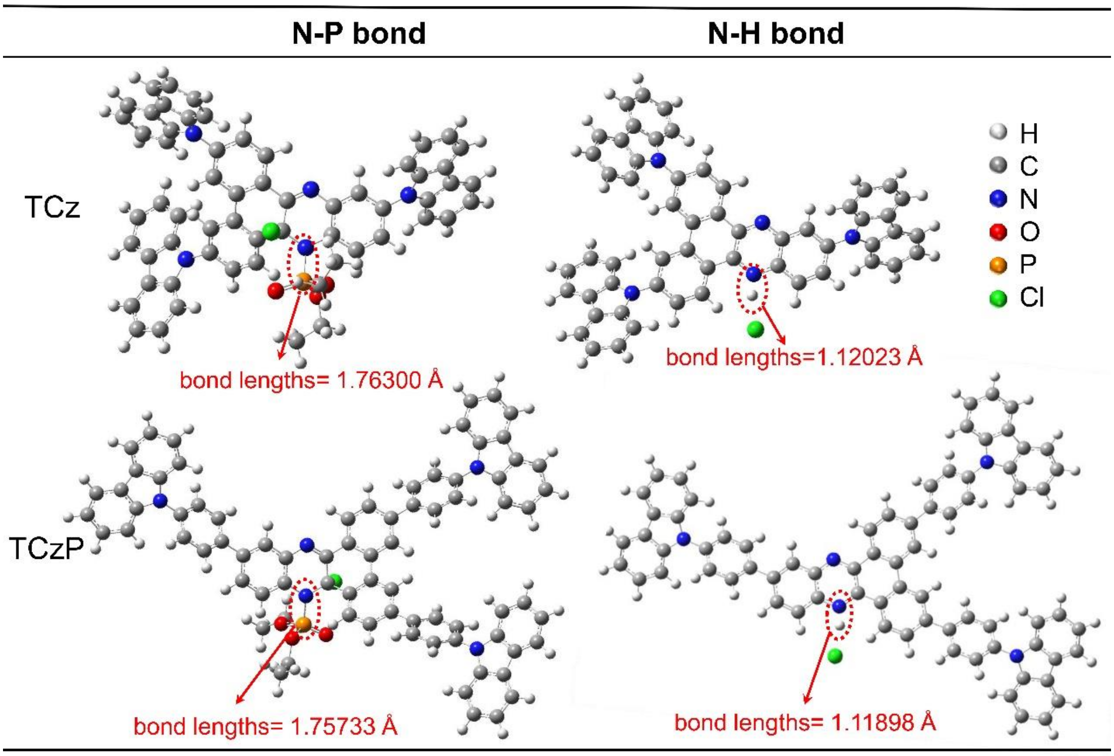

Supplementary Figure 14. Bond lengths and of N-H and N-P bonds of TCz and TCzP in angstroms are given at the b3lyp/6-31g(d,p).

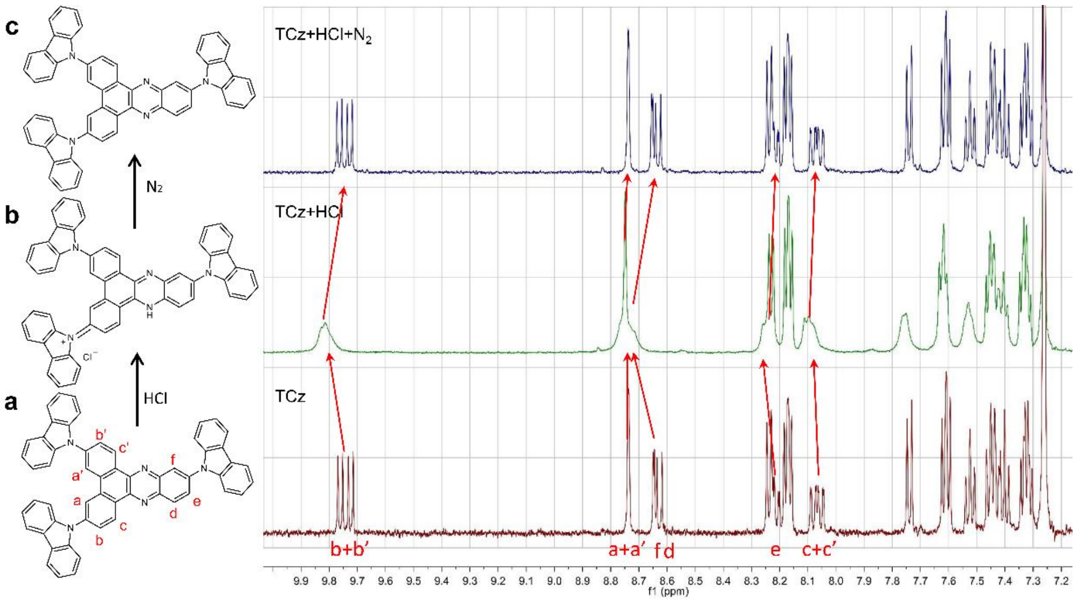

Supplementary Figure 15. ^{1}H NMR mechanism analysis. a the ^{1}H NMR of TCz; **b** the ^{1}H NMR of TCz was protonated with excess HCl vapors for 0.5 h; **c** the ^{1}H NMR of protonated TCz was blown with N_2 at a flow rate of 700 mL/min for 0.5 h.

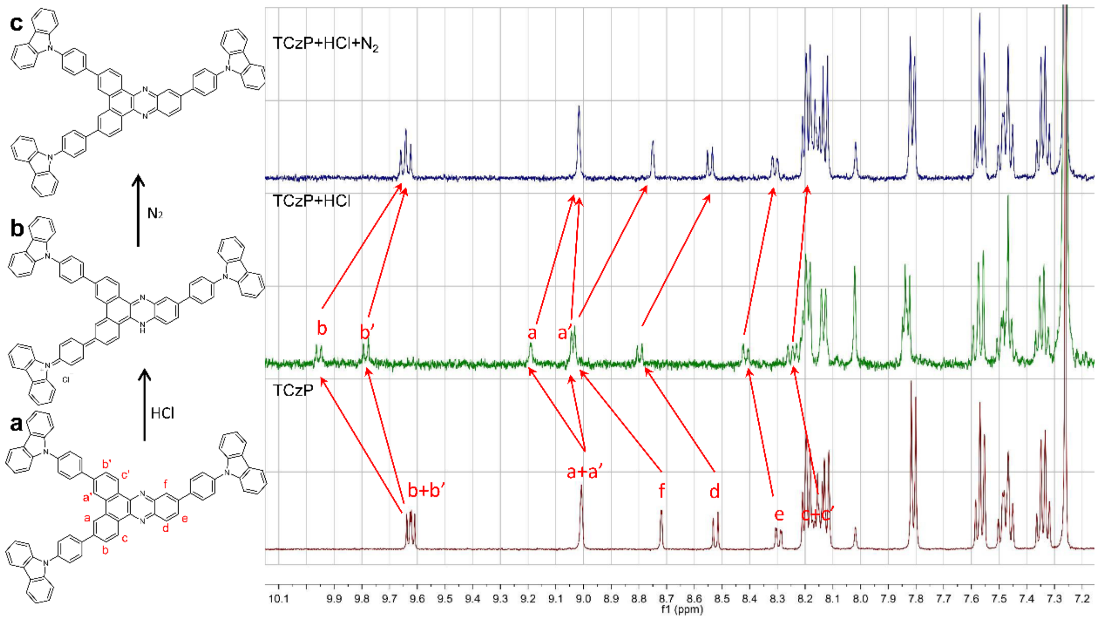

Supplementary Figure 16. ^{1}H NMR mechanism analysis. a the ^{1}H NMR of TCzP; **b** the ^{1}H NMR of TCzP was protonated with excess HCl vapors for 0.5 h; **c** the ^{1}H NMR of protonated TCzP was blown with N_2 at a flow rate of 700 mL/min for 0.5 h.

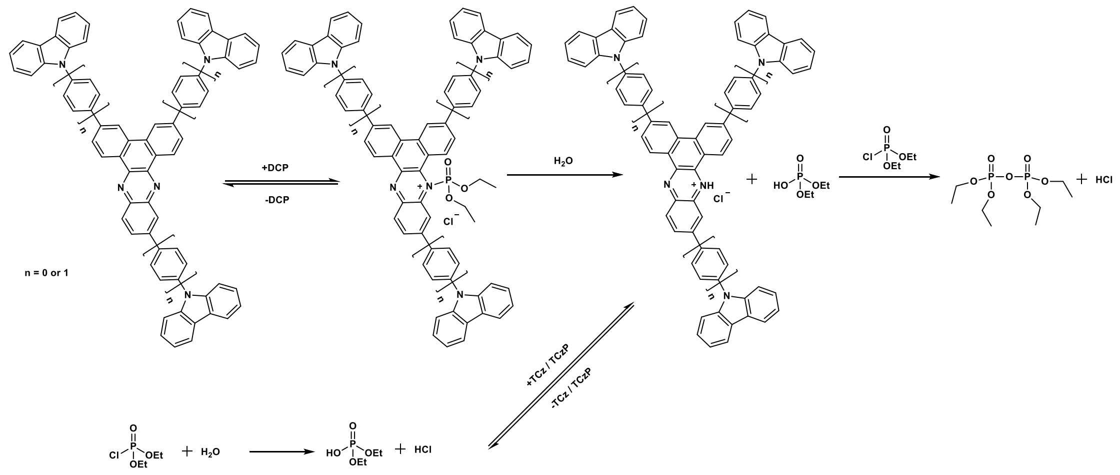

Supplementary Figure 17. Sensing mechanism.

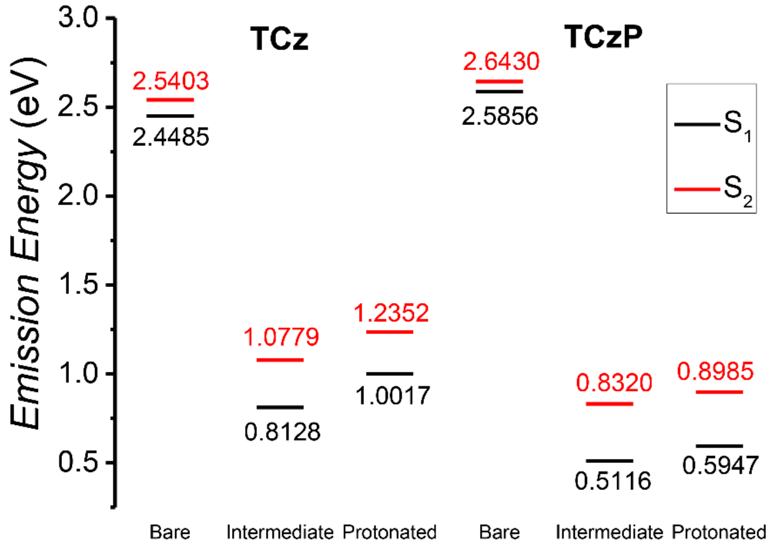

Supplementary Figure 18. Excited state energy. Calculated bared vs protonated emission energy of TCz and TCzP.

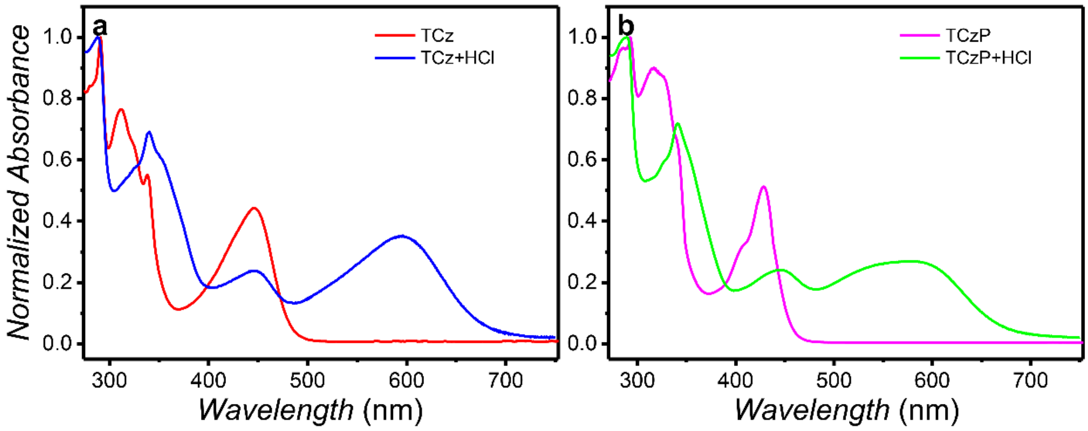

Supplementary Figure 19. UV-Vis absorption spectra. UV-Vis absorption spectra of TCz (**a**) and TCzP (**b**) in DCM (DCM is stirred with water to stand the lower layer liquid after stratification) before and after the addition of HCl (5 μL, 12 M).

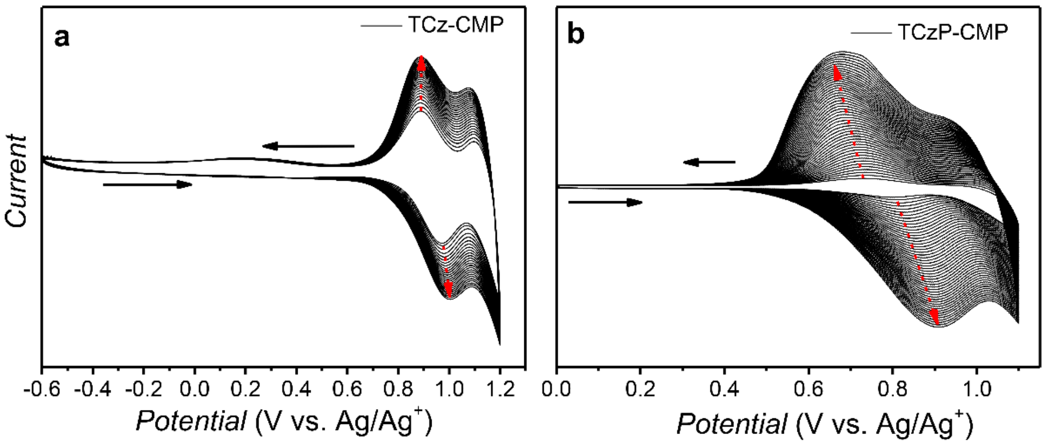

Supplementary Figure 20. Electrochemical polymerization. a Cyclic voltammetry curve for the preparation of TCz-CMPs films. (scanning cycles: 50 cycles; scanning rate: 100 mV/s; scanning potentials: -0.6-1.2 V) **b** Cyclic voltammetry curve for the preparation of TCzP-CMPs films. (scanning cycles: 100 cycles; scanning rate: 200 mV/s; scanning potentials: 0-1.1 V)

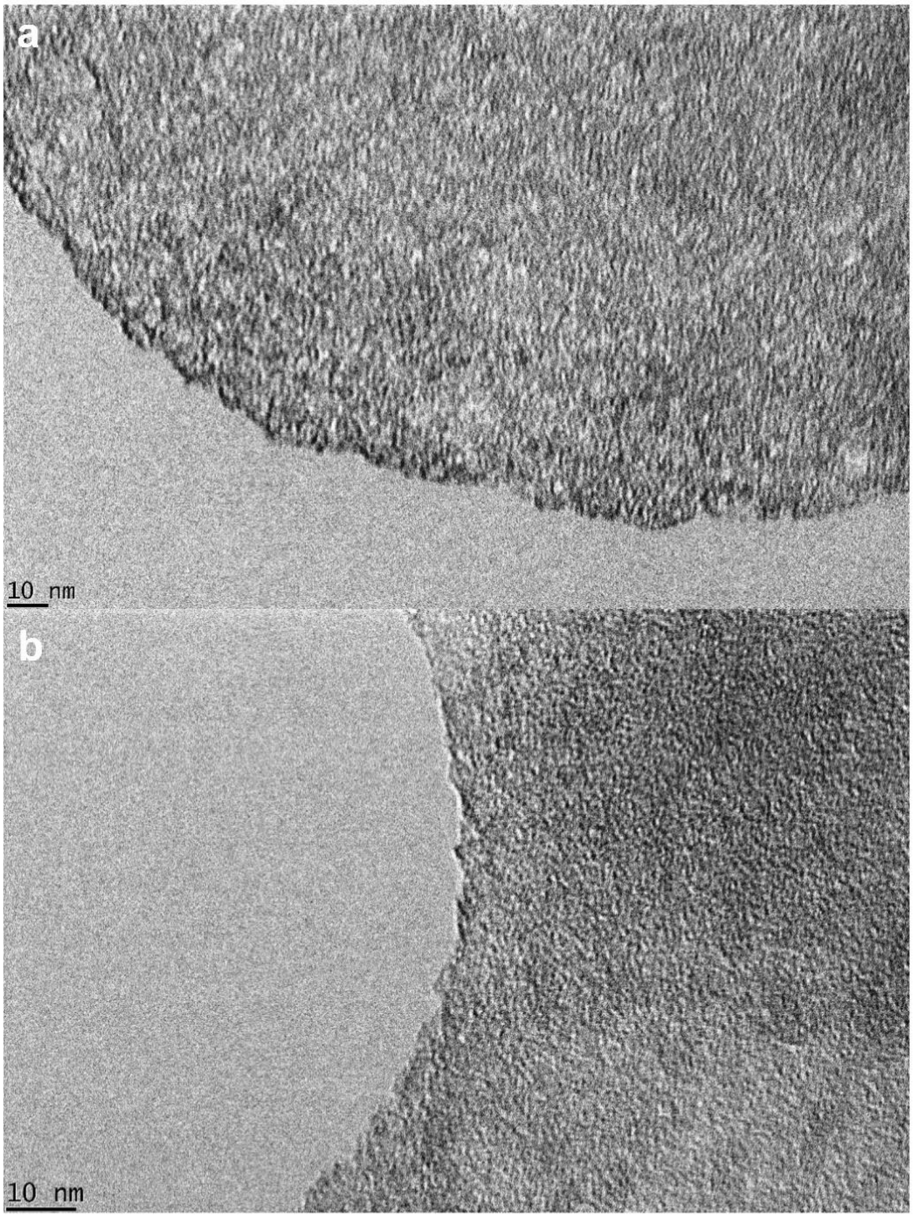

Supplementary Figure 21. TEM images. TEM images of TCz-CMP films (**a**) and TCzP-CMP films (**b**).

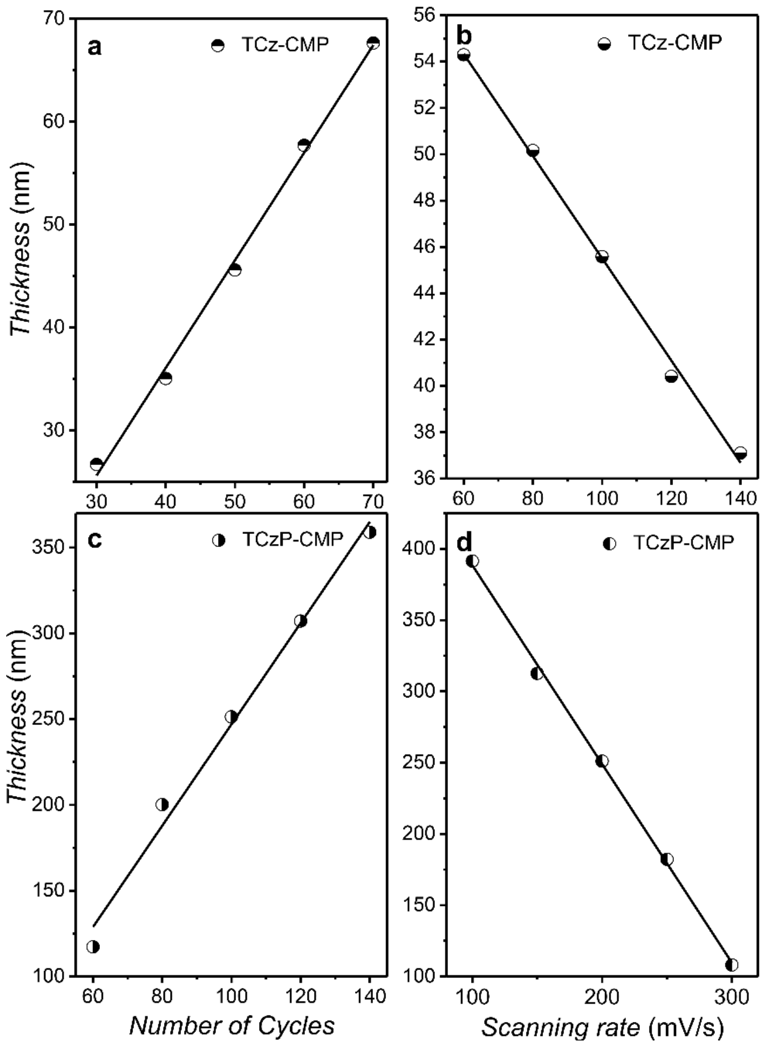

Supplementary Figure 22. The optimization of film thickness. a The linear relationship between the thickness of the TCz-CMP films and the scanning cycles when the scanning rate is 100 mV/s. **b** The thickness of the TCz-CMP films decreases as the scanning rate increases when the scanning cycles are 50 cycles. **c** The linear relationship between the thickness of the TCzP-CMP films and the scanning cycles when the scanning rate is 200 mV/s. **d** The thickness of the TCzP-CMP film decreases as the scanning rate increases when the scanning cycles are 100 cycles.

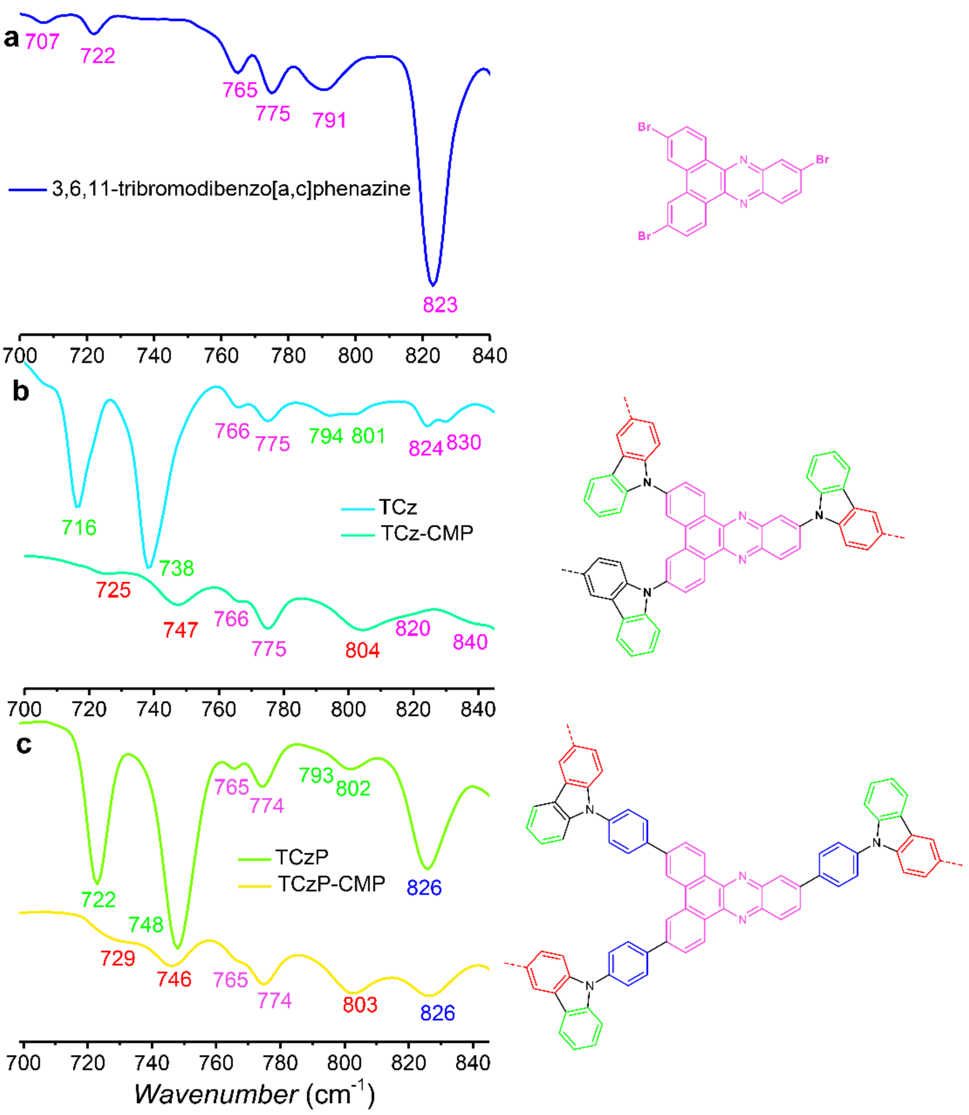

Supplementary Figure 23. FT-IR spectroscopy analysis. a (Left) FT-IR spectrum of 3,6,11-tribromodibenzo [a,c] phenazine and (Right) their peak assignment. **b** (Left) FT-IR spectrum of TCz-CMP (red curve) and monomer (black curve), and (Right) their peak assignment. **c** (Left) FT-IR spectrum of TCzP-CMP (red curve) and monomer (black curve), and (Right) their peak assignment.

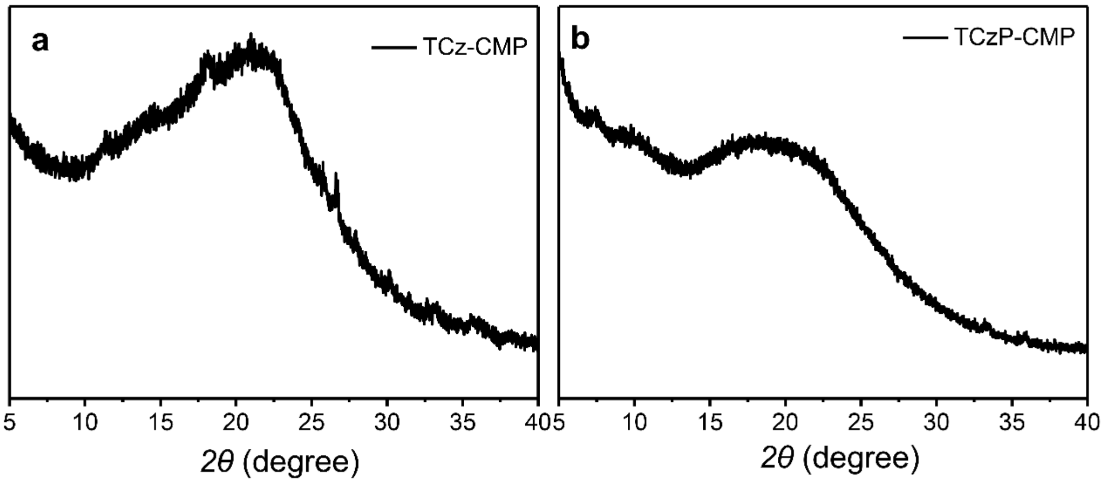

Supplementary Figure 24. XRD pattern of the TCz-CMP and TCzP-CMP. The powder X-ray diffraction (XRD) patterns of TCz-CMP (**a**) and TCzP-CMP (**b**) demonstrate a broad and dispersion peak within the 2*θ* range of 5-40 °.

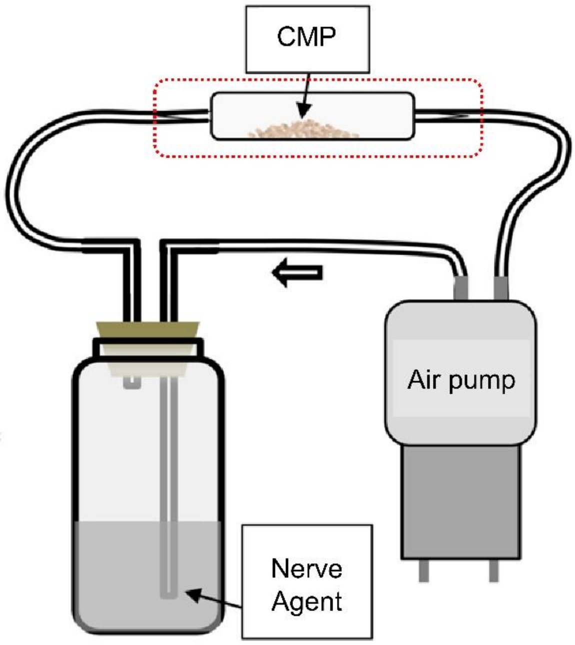

Supplementary Figure 25. Diagram of CMP adsorption test. The part within the red dotted line can be removed for weighing. Among them, the volume of CMP sample compartment is 5 mL, nerve agent sample bottle is 20 mL (containing 3 mL liquid DCP). Kamoer EDLP600-D12 diaphragm pump was used to inject DCP vapors to sample compartment, and the specific specifications of pump are as follows: voltage 12 V, rated current 300 mA, rated power is 5 W, and the flow about 600 mL/min.

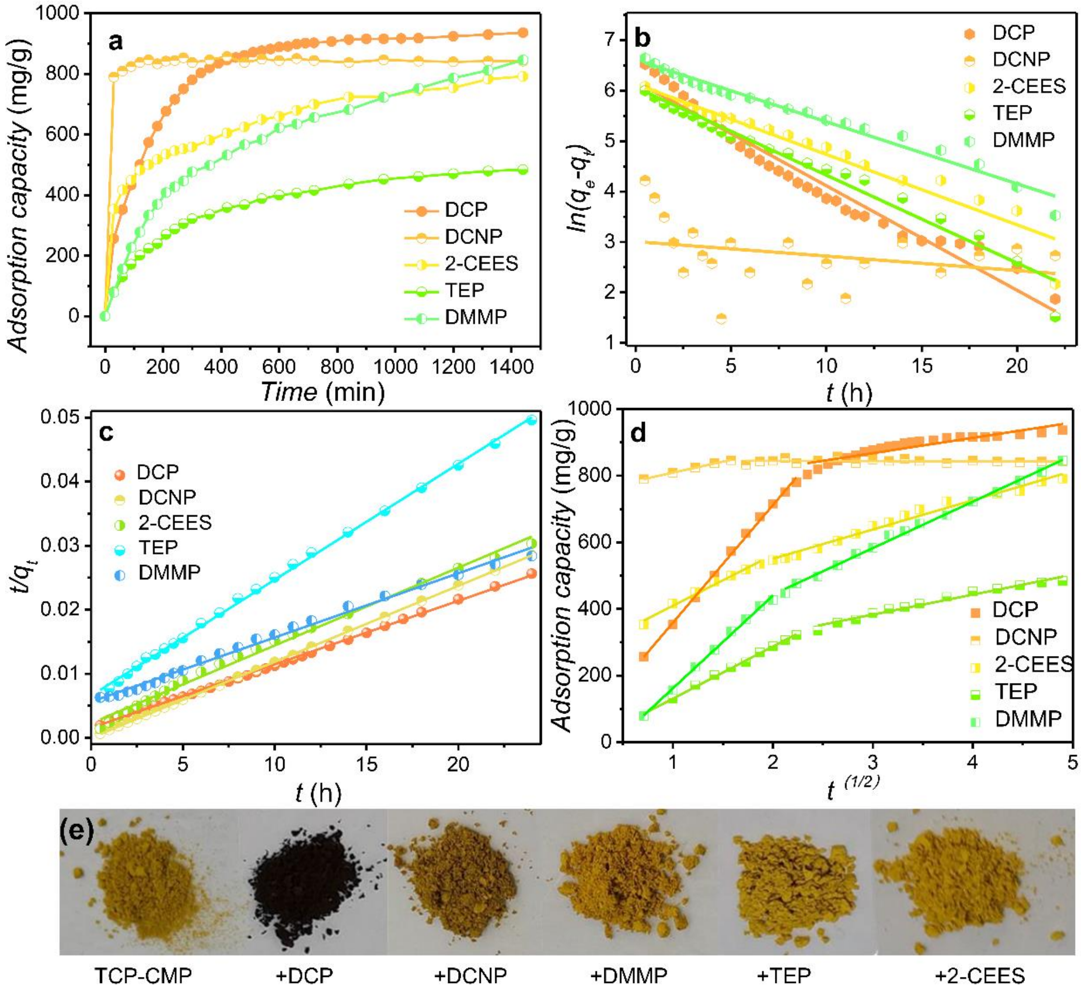

Supplementary Figure 26. CMP adsorption of other toxic agent simulants. a Adsorption kinetics of five chemical warfare agent simulants by TCzP-CMP; **b** PFO model; **c** PSO model; **d** intraparticle diffusion model; **e** Photos of TCzP-CMP before and after the adsorption of five CWA simulants for 24 h. The original adsorption data is in **Source Data**.

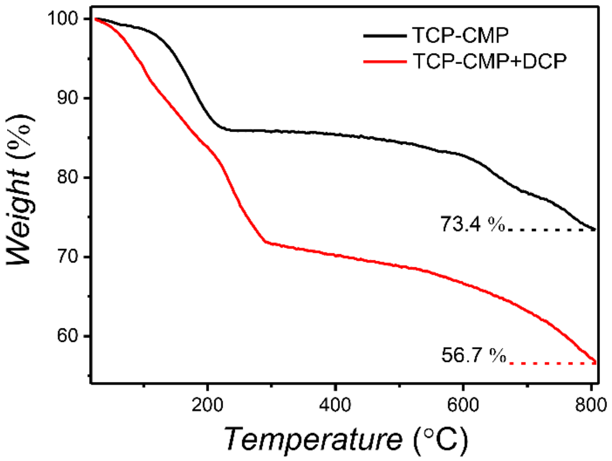

Supplementary Figure 27. The thermogravimetric analysis (TGA) of TCzP-CMP was performed in the absence and presence of DCP (heating rate 10 ℃/min).

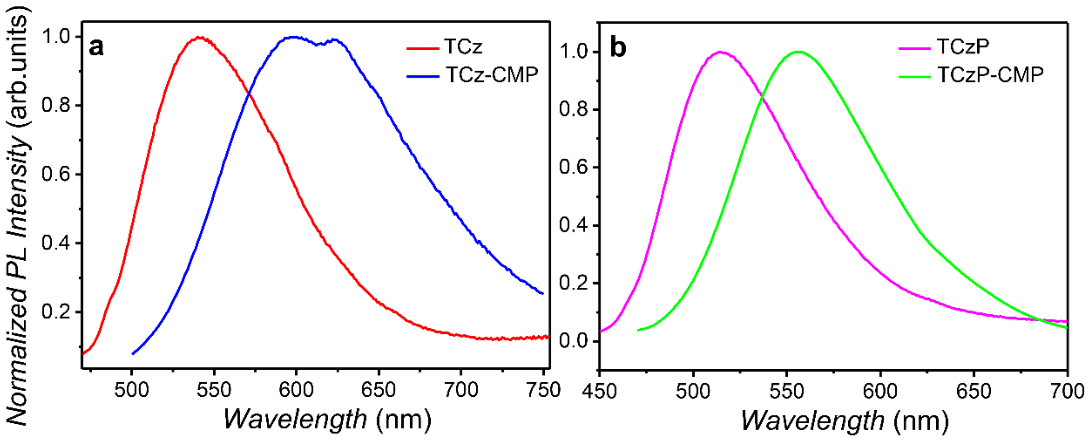

Supplementary Figure 28. The fluorescent properties. Fluorescence spectra of spin-coated films and CMP films based on TCz (**a**) and TCzP (**b**).

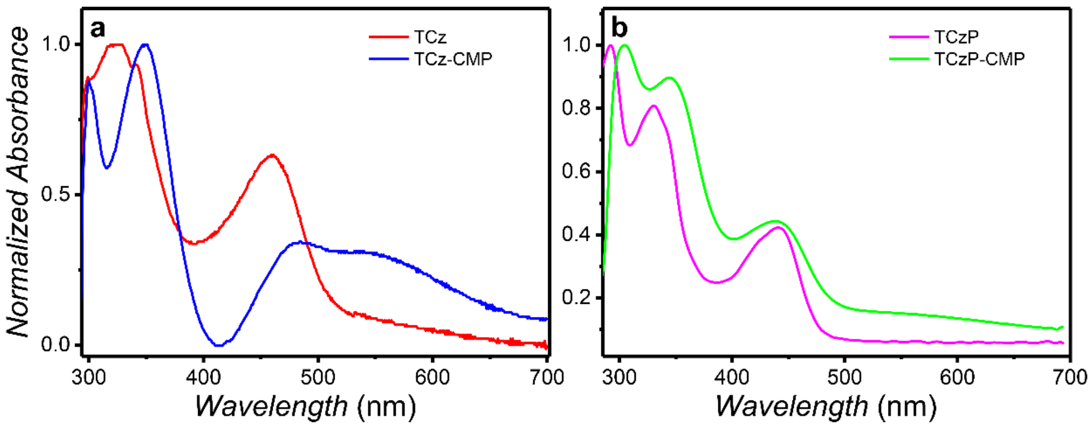

Supplementary Figure 29. UV-Vis absorption spectra of spin-coated films and CMP films of TCz (a) and TCzP (b).

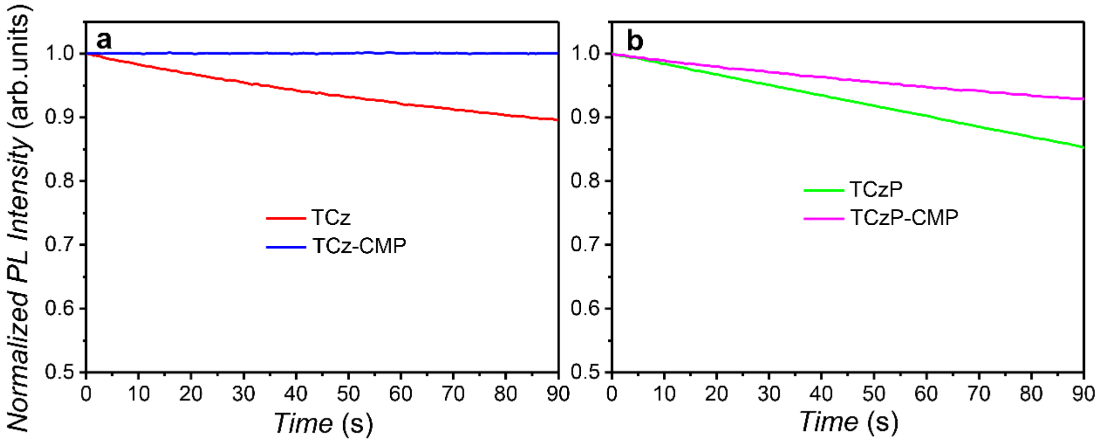

Supplementary Figure 30. Fluorescence intensity of spin-coated films and CMP films based on TCz (a) and TCzP (b) as the function of excitation time in air.

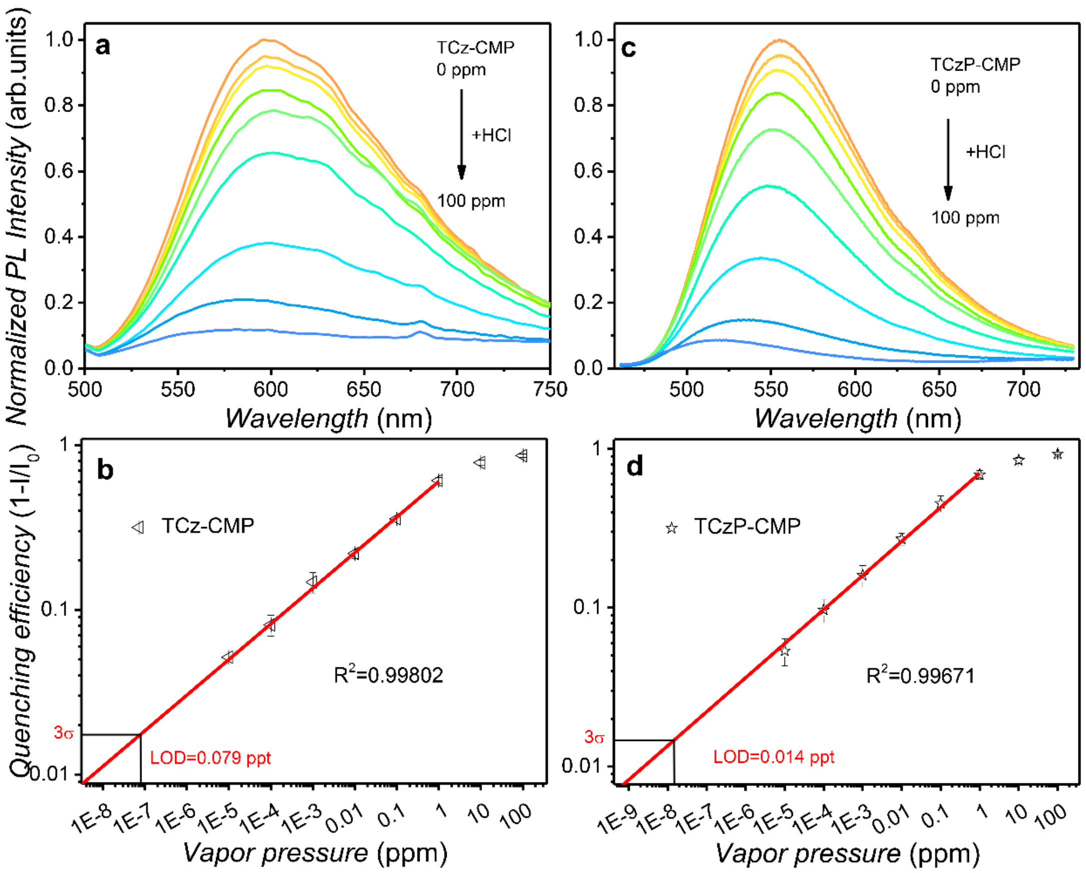

Supplementary Figure 31. **Titration experiment of HCl. a** The fluorescence intensity of TCz CMP films to HCl vapors (0-100 ppm). **b** The quenching efficiency of TCz CMP films exposed to HCl vapors. **c** The fluorescence intensity of TCzP CMP films to HCl vapors (0-100 ppm). **d** The quenching efficiency of TCzP CMP films exposed to HCl vapors. Among them, the thickness of TCz-CMP and TCzP-CMP films are 45 nm and 250 nm, respectively. Error bars stand for standard deviation (n = 3).

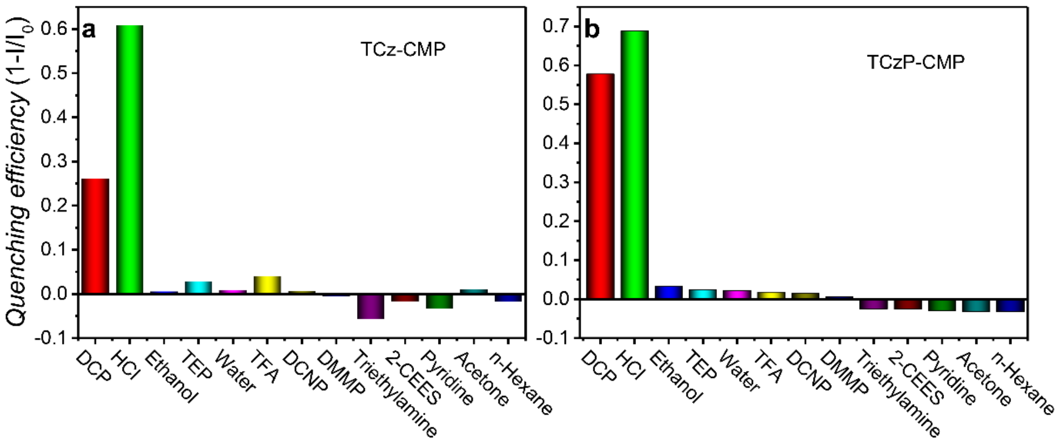

Supplementary Figure 32. Interference test. The quenching efficiency of TCz-CMP (**a**) and TCzP-CMP (**b**) films to DCP (acid-free; 1.32 ppm), HCl (1 ppm), ethanol (780 ppm), TEP (99 ppm), water (32000 ppm), TFA (13 ppm), DCNP (2 ppm), DMMP (200 ppm), triethylamine (1090 ppm), 2-CEES (38 ppm), pyridine (25 ppm), acetone (816 ppm) and n-hexane (21 ppm) vapors, respectively.

Supplementary Table

Supplementary Table 1. The photoluminescence efficiency, lifetime, radiation and non-radiation transition rate of compounds in four solvents.

Solvents	TCz				TCzP			
	$\eta(\%)^a$	τ^b	k_r^c	k_{nr}^d	$\eta(\%)^a$	τ^b	k_r^c	k_{nr}^d
n-Hexane	21.71	1.40	15.46	55.76	11.42	0.56	20.46	158.69
Isopropyl ether	27.21	1.38	19.72	52.75	31.92	1.37	23.30	49.69
Ether	36.13	1.77	20.45	36.15	33.99	1.55	21.99	42.70
Dichloromethane	59.01	7.93	9.56	6.64	82.50	6.64	13.67	2.90
Acetone	23.36	4.82	4.85	15.91	21.98	4.67	4.68	16.60

[a] photoluminescence efficiency; [b] lifetime (ns); [c] radiation transition rate (10^7 s^{-1}) and [d] non-radiative transition rate (10^7 s^{-1})

Supplementary Table 2. Solvatochromic UV-PL data for Lippert-Mataga model.

Solvents	Δf	TCz			TCzP		
		ν_a (nm)	ν_f (nm)	ν_a - ν_f (cm^{-1})	ν_a (nm)	ν_f (nm)	ν_a - ν_f (cm^{-1})
n-Hexane	0.0012	453	465	569.68	424	446	1163.38
Triethylamine	0.048	453	474	978.01	428	451	1191.54
Butyl ether	0.096	453	484	1413.90	428	464	1812.76
Isopropyl ether	0.145	451	493	1888.97	425	459	1742.92
Ether	0.167	443	502	2653.04	425	471	2297.99
Ethyl acetate	0.2	443	530	3705.44	428	506	3601.64
Tetrahydrofuran	0.21	446	537	3799.55	430	522	4098.73
Dichloromethane	0.217	445	553	4388.73	429	548	5061.85
Dimethylformamide	0.276	446	599	5727.03	434	626	7067.03
Acetone	0.284	428	579	6093.33	429	591	6389.55
Acetonitrile	0.305	438	595	6024.33	427	636	7695.93

Supplementary Table 3. Summary of the reported LOD of DCP from previous work.

Sensing Materials	LOD	Solution (S) or Vapor (V)	Reference
TCz	1.9 ppb (acid-containing)	V	This work
TCzP	1 ppb (acid-containing)	V	This work
TCz	69 ppb (acid-free)	V	This work
TCzP	6.9 ppb (acid-free)	V	This work
TCz-CMP	0.14 ppt (acid-containing)	V	This work
TCzP-CMP	0.032 ppt (acid-containing)	V	This work
TCz-CMP	21 ppt (acid-free)	V	This work
TCzP-CMP	2.5 ppt (acid-free)	V	This work
TPA-9AC	0.15 ppb	V	1
B1-SBA	15 $\mu g/m^3$	V	2
P1	2 ppm	V	3
1D PC	4 ppm	V	4
Sample 1	15 ppb	V	5
Sample 1	4 ppb	V	6
Sample 1-2	8 ppb	V	7
TPOD	0.14 µM	V	8
DPA-TPE-Py	1.82 ppb	V	9
PTS	10.4 nM	V/S	10
TBPY-TPA	2.6 ppb	V	11
Sample 2	0.14 ppb	V	12
TOP-I	1.2 ppb	V	13
PAC-1	28 ppb	V	14
PY-0PD	--	V	15
FLA	13 ppm	V	16
T1	0.8 ppb	V	17
BT-OH	0.186 µM	S/V	18
P1	2.3 nM/0.7 ppb	S/V	19
TPIM	10^{-8} M	S/V	20
NA570	5 µM	S	21
Sample 3	8 nM	S/V	22
CYD	18.86 nM	S	23
PDAC	88 nM	S	24
FLA	1 µM	S	25
Sample 8	2.6 µM	S	26
RB-AE	25 ppm	S	27
S-I-SBA	90.8 pM	S	28
RDS	9.66×10^{-9} M	S	29
PQ	7 ppm	S	30
AIL-4	131.5 ppb	S	31
Sample 1	0.17 ppm	S	32
AQmol-1	0.18 µM	S	33

AQmol-2	0.16 μM	S	33
Sample 1	1.87 ppb	S	34
$CoumNMe_2$	4.4×10^{-8} M	S	35
B–SAL–OXIME	900 μM	S	36
Sample 2	1.72 μM	S	37
Sample 1	0.065 μM	S	38
Sample 2	2.1 μM	S	38
NA-p3	21 nM	S	39
m-Py-BOD	3.36 μM	S	40
probe 1	0.136 nM	S	41

Supplementary Table 4. Summary of the reported LOD of DCP and HCl from this work.

Sensing Materials	LOD		
	DCP		HCl
	Air(acid-containing)	N2 (acid-free)	
TCz	1.9 ppb	69 ppb	84 ppt
TCzP	1.0 ppb	6.6 ppb	140 ppt
TCz-CMP	0.14 ppt	21 ppt	0.079 ppt
TCzP-CMP	0.032 ppt	2.5 ppt	0.014 ppt

Supplementary Table 5. Kinetic parameters of pseudo-first and pseudo-secondary models of TCz-CMP and TCzP-CMP adsorption of CWA simulants.

Material	Compound	$q_{e,exp}$	PSO			PFO		
			$q_{e,cal}$	K_2	R^2	$q_{e,cal}$	K_1	R^2
TCz-CMP	DCP	78.4	81.8	0.0118	0.99583	82.1	0.1295	0.7874
TCzP-CMP	DCP	936.2	976.5	0.000993	0.99869	942.8	0.2072	0.9558
	DCNP	842.1	875.8	0.00119	0.99994	1690.4	0.0287	0.0321
	2-CEES	791.7	824.5	0.00122	0.99345	820.5	0.1395	0.93351
	TEP-2	484.0	506.0	0.00181	0.99332	491.6	0.1741	0.968
	DMMP	846.1	885.9	0.001	0.9986	892.9	0.1229	0.975

Supplementary Table 6. Intra-particle diffusion model parameters of TCz-CMP and TCzP-CMP adsorption of CWA simulants.

Material	Compound	Step1			Step2		
		C_1	K_{P1}	R_{P1}^2	C_2	K_{P2}	R_{P2}^2
TCz-CMP	DCP	26.90	15.47	0.97522	73.12	1.25	0.46339
TCzP-CMP	DCP	6.13	325.84	0.99745	729.84	45.97	0.84481
	2-CEES	257.00	152.73	0.9809	375.40	87.74	0.98036
	DCNP	743.13	66.12	0.99811	845.89	-0.44	-0.0566
	TEP	-25.79	156.63	0.9974	203.16	59.97	0.97245
	DMMP	-116.54	278.40	0.9964	164.32	139.46	0.99611

Supplementary Table 7. The adsorption capacity of TCzP-CMP and activated carbon on CWA simulants.

Material	Adsorption capacity (mg/g)				
	DCP	DCNP	DMMP	TEP	2-CEES
Activated carbon	319.8	253.0	475.6	283.0	455.3
TCzP-CMP	936.2	842.1	846.1	484.0	791.7

Supplementary Notes

Supplementary Note 1. Radiation form calculation. According to the following formulas, the radiative and non-radiative transition rates of the compound in various states are obtained[42]:

$$k_r = \frac{\Phi_f}{\tau} \qquad \text{(Supplementary Equation 1)}$$

$$\Phi_f = \frac{k_r}{k_r + k_{nr}} \qquad \text{(Supplementary Equation 2)}$$

Where Φ_f is the luminous efficiency, τ is the lifetime, k_r is the radiative transition rate, and k_{nr} is the non-radiative transition rate.

Supplementary Note 2. Solvatochromic UV-PL data for Lippert-Mataga model. The following Lippert-Mataga solvation model was used in the experiment[43]:

$$hc(\nu_a - \nu_f) = hc(\nu_a^0 - \nu_f^0) + \frac{2(\boldsymbol{\mu_e} - \boldsymbol{\mu_g})^2}{a_0^3} f(\varepsilon, n) \qquad \text{(Supplementary Equation 3)}$$

Where h is the Planck constant; $(\nu_a - \nu_f)$ is the Stokes shift; $(\nu_a^0 - \nu_f^0)$ is the Stokes shift when the polarity factor f=0; is the polarity factor of the solvent, which is related to the vacuum dielectric constant ε and the refractive index n,$f = \frac{\varepsilon-1}{2\varepsilon+1} + \frac{n^2-1}{2n^2+1}$; a_0 is the Angsag radius of the molecule, $a_0 = (\frac{3M}{4N\pi d})^{1/3}$; where M is the molar mass of the molecule; N is the Avogadro constant; d is the relative density of the solvent; π is the ratio of the circumference of a circle to the diameter; $\boldsymbol{\mu_e}$ and $\boldsymbol{\mu_g}$ are the dipoles of the excited state and ground state, respectively. By drawing the slope of the Stokes shift and the solvent polarity factor, the dipole moment of the excited state can be calculated.

Supplementary Note 3. Preparation of spin-coated films. The spin-coated films were prepared by a KW-4A spin-coating. TCz (5×10^{-5} M) or TCzP (4×10^{-4} M) solution was dropped on the quartz glass plate, and setting the rotation speed of the spin coater to 3000 r/min, and the rotation time is 15 s. The obtained spin-coated film was then dried in a vacuum drying oven for 1 h.

Supplementary Note 4. Fluorescence detection of DCP vapors. (1) Common method: 2 mL DCP was injected into a 40 mL jar under the air atmosphere and sealed the jar for 48 h at room temperature. Then, a small amount of saturated DCP vapors was diluted to obtain different concentrations of DCP vapors (acid-containing) for LOD detection[17]. (2) To eliminate hydrolysate HCl, excessive K_2CO_3 was added into DCP liquid and the saturated DCP vapors were obtained by sealing the DCP liquid containing K_2CO_3 in a high-purity N_2 bag for 48 h at room temperature. And then the LOD was measured by quenching fluorescence films with different concentrations of DCP vapors (acid-free) obtained by diluting the saturated DCP vapor with N_2[44]. In all the LOD test experiments, the fluorescent films were exposed to the vapors for 15 s.

Supplementary Note 5. Reversibility verification of the response mechanism:

In general, the chemical bonds become less stable as their length increases. The bond lengths of the N-P bonds generated by the reaction of TCz and TCzP with DCP and the N-H bond lengths of their protonated products were calculated by density functional theory (DFT). As shown in **Supplementary Figure 14**, the bond length of the N-H bond (1.12023 and 1.11898 Å) is significantly smaller than that of the N-P bond (1.76300 and 1.75733 Å), and the stability of the N-H bond is much greater than that of the N-P bond. Since N-P intermediates are unstable and difficult to be captured, the protonated products of TCz and TCzP were took as the research objects.

First, the powders of TCz and TCzP were respectively placed in excess HCl vapors to react for 0.5 h, and a part of their protonated products were taken for the ^{1}H NMR spectra test (**Supplementary Figure 15b, 16b**). Then, their remaining protonated products were respectively blown with N_2 at a flow rate of 700 mL/min for 0.5 h, and their ^{1}H NMR spectra were tested again (**Supplementary Figure 15c, 16c**).

Compare with their ^{1}H NMR spectra before protonation (**Supplementary Figure 15a, 16a**). It can be seen that the chemical shifts of H on Dibenzo[*a,c*]phenazine (DPPZ) after protonation shift downfield, which is a typical protonation feature. Excitingly, the ^{1}H NMR spectra of TCz and TCzP recovered again after N_2 blowing. The more stable N-H bond can be recovered, which is enough to show that the N-P bond is completely possible to break after N_2 blowing at room temperature.

Supplementary Note 6. Theoretical Calculations. The density functional theory (DFT) calculations and the natural transition orbit (NTO) analysis are carried out using a Gaussian 09 D.01 Package. The ground state conformations are optimized using opt/b3lyp/6-31 g (d, p) method, and the excited state properties are calculated using td-b3lyp/6-31 g (d, p) method.

Supplementary Note 7. Nitrogen adsorption/desorption measurements. Nitrogen adsorption/desorption measurements were performed on an ASAP 2020 plus at 77 K. The CMP samples were degassed under vacuum at 110 °C for 12 h before measurements. The Brunauer-Emmet-Teller (BET) surface area of CMP films can be calculated based on the adsorption-desorption isotherms. The pore-size distribution profile was obtained by the nonlocal density functional theory (NL-DFT) method.

Supplementary Note 8. Accuracy verification of the adsorption equipment

In order to verify the results of this experiment, the thermogravimetric analysis (TGA) of TCzP-CMP was performed in the absence and presence of DCP (heating rate 10 °C/min)[45]. As **Supplementary Figure 27** shown, the char residue of pure TCzP-CMP is 73.38%, while the char residue of TCzP-CMP (adsorption capacity 327 mg/g) after adsorption of DCP for 1 h using the device shown in **Supplementary Figure 25** under the same conditions is 56.71%.

After calculation according to the formula:

$$q_e = \frac{1 - \frac{a}{b}}{\frac{a}{b}} \qquad \text{(Supplementary Equation 4)}$$

q_e: adsorption capacity of TCzP-CMP to DCP; a: the char residue after TCzP-CMP adsorption of DCP (327 mg/g); a: the char residue of pure TCzP-CMP.

The adsorption capacity of TCzP-CMP by TGA is 294 mg/g, and the error range compared with our test method is 10.22%, which may be due to the volatilization of DCP during sample storage. Therefore, the measurement provided in our manuscript is solid.

Supplementary References:

1. Li X, et al. Visualization of ultrasensitive and recyclable dual-channel fluorescence sensors for chemical warfare agents based on the state dehybridization of hybrid locally excited and charge transfer materials. *Anal. Chem.* **91**, 10927-10931 (2019).

2. Climent E, et al. A rapid and sensitive strip-based quick test for nerve agents Tabun, Sarin, and Soman using BODIPY-Modified silica materials. *Chem. - Eur. J.* **22**, 11138-11142 (2016).

3. Annisa TN, Jung S-H, Gupta M, Bae Jy, Park JM, Lee H-i. A reusable polymeric film for the alternating colorimetric detection of a nerve agent mimic and ammonia vapor with sub-parts-per-million sensitivity. *ACS Appl. Mater.Interfaces* **12**, 11055-11062 (2020).

4. Jung S-H, et al. Chromophore-Free photonic multilayer films for the ultra-sensitive colorimetric detection of nerve agent mimics in the vapor phase. *Sens. Actuators, B* **323**, 128698 (2020).

5. Liu X, et al. Sensitive detection of a nerve-agent simulant through retightening internanofiber binding for fluorescence enhancement. *Anal. Chem.* **90**, 1498-1501 (2018).

6. Sun C, et al. Fast and ultrasensitive detection of a nerve agent simulant using carbazole-based nanofibers with amplified ratiometric fluorescence responses. *Anal. Chem.* **90**, 7131-7134 (2018).

7. Xiong W, Gong Y, Che Y, Zhao J. Sensitive discrimination of nerve agent and sulfur mustard simulants using fluorescent coassembled nanofibers with Förster resonance energy transfer-enhanced photostability and emission. *Anal. Chem.* **91**, 1711-1714 (2019).

8. Kammer M, et al. Rapid quantification of two chemical nerve agent metabolites in serum. *Biosens. Bioelectron.* **131**, 119-127 (2019).

9. Huang S, Wu Y, Zeng F, Sun L, Wu S. Handy ratiometric detection of gaseous nerve agents with AIE-fluorophore-based solid test strips. *J. Mater. Chem. C* **4**, 10105-10110 (2016).

10. Huo B, et al. "Covalent-Assembly"-based fluorescent probe for detection of a nerve-agent mimic (DCP) via lossen rearrangement. *Anal. Chem.* **91**, 10979-10983 (2019).

11. Yao J, et al. Concise and efficient fluorescent probe via an intromolecular charge transfer for the chemical warfare agent mimic diethylchlorophosphate vapor detection. *Anal. Chem.* **88**, 2497-2501 (2016).

12. Fu Y, et al. Simple and efficient chromophoric-fluorogenic probes for diethylchlorophosphate vapor. *ACS Sens.* **3**, 1445-1450 (2018).

13. Xu W, et al. Aggregation state reactivity activation of intramolecular charge transfer type

fluorescent probe and application in trace vapor detection of Sarin mimics. *ACS Sens.* **1**, 1054-1059 (2016).

14. Zhu R, Azzarelli JM, Swager TM. Wireless hazard badges to detect nerve-agent. *Angew. Chem. Int. Ed.* **55**, 9662-9666 (2016).

15. Zhou X, Zeng Y, Liyan C, Wu X, Yoon J. A fluorescent sensor for dual-channel discrimination between phosgene and a nerve-gas mimic. *Angew.Chem. Int. Ed.* **55**, 4729-4733 (2016).

16. Bencic-Nagale S, Sternfeld T, Walt DR. Microbead chemical switches: An approach to detection of reactive organophosphate chemical warfare agent vapors. *J. Am. Ceram. Soc.* **128**, 5041-5048 (2006).

17. Zheng P, et al. A simple organic multi-analyte fluorescent prober: One molecule realizes the detection to DNT, TATP and Sarin substitute gas. *J. Hazard. Mater.*, **409**, 124500 (2021).

18. Hu X, Zeng H, Chen T, Yuan H-Q, Zeng L, Bao G-M. Fast and visual detection of a chemical warfare agent mimic using a simple, effective and portable chemodosimeter. *Sens. Actuators, B* **319**, 128282 (2020).

19. Zheng P, Abdurahman A, Liu G, Liu H, Zhang Y, Zhang M. An instantaneously-responded, ultrasensitive, reutilizable fluorescent probe to sarin substitute both in solution and in gas phase. *Sens. Actuators, B* **322**, 128611 (2020).

20. Aich K, Das S, Gharami S, Patra L, Kumar Mondal T. Triphenylamine–benzimidazole based switch offers reliable detection of organophosphorus nerve agent (DCP) both in solution and gaseous state. *New J. Chem.* **41**, 12562-12568 (2017).

21. Lei Z, Yang Y. A concise colorimetric and fluorimetric probe for Sarin related threats designed via the "Covalent-Assembly" approach. *J. Am. Ceram. Soc.* **136**, 6594-6597 (2014).

22. Cai Y-C, Li C, Song Q-H. Fluorescent chemosensors with varying degrees of intramolecular charge transfer for detection of a nerve agent mimic in solutions and in vapor. *ACS Sens.* **2**, 834-841 (2017).

23. Ali SS, et al. A chromogenic and ratiometric fluorogenic probe for rapid detection of a nerve agent simulant DCP based on a hybrid hydroxynaphthalene-hemicyanine dye. *Org. Biomol. Chem.* **15**, 5959-5967 (2017).

24. Zeng L, Zeng H, Jiang L, Wang S, Hou J-T, Yoon J. A single fluorescent chemosensor for simultaneous discriminative detection of gaseous phosgene and a nerve agent mimic. *Anal. Chem.* **91**, 12070-12076 (2019).

25. Singh VV, et al. Micromotor-based on–off fluorescence detection of sarin and soman simulants.

Chem. Commun. **51**, 11190-11193 (2015).

26. Wild A, Winter A, Hager MD, Schubert US. Fluorometric, water-based sensors for the detection of nerve gas G mimics DMMP, DCP and DCNP. *Chem. Commun.* **48**, 964-966 (2012).

27. Wu X, Wu Z, Han S. Chromogenic and fluorogenic detection of a nerve agent simulant with a rhodamine-deoxylactam based sensor. *Chem. Commun.* **47**, 11468-11470 (2011).

28. Climent E, et al. Determination of the chemical warfare agents Sarin, Soman and Tabun in natural waters employing fluorescent hybrid silica materials. *Sens. Actuators, B* **246**, 1056-1065 (2017).

29. Heo G, Manivannan R, Kim H, Son Y-A. Liquid and gaseous state visual detection of chemical warfare agent mimic DCP by optical sensor. *Dyes Pigm.* **171**, 107712 (2019).

30. Jo S, Kim D, Son S-H, Kim Y, Lee TS. Conjugated poly(fluorene-quinoxaline) for fluorescence imaging and chemical detection of nerve agents with its paper-based strip. *ACS Appl. Mater. Interfaces* **6**, 1330-1336 (2014).

31. Chen C-Y, Li K-H, Chu Y-H. Reaction-Based detection of chemical warfare agent mimics with affinity ionic liquids. *Anal. Chem.* **90**, 8320-8325 (2018).

32. Xuan W, Cao Y, Zhou J, Wang W. A FRET-based ratiometric fluorescent and colorimetric probe for the facile detection of organophosphonate nerve agent mimic DCP. *Chem. Commun.* **49**, 10474-10476 (2013).

33. Dagnaw FW, Feng W, Song Q-H. Selective and rapid detection of nerve agent simulants by polymer fibers with a fluorescent chemosensor in gas phase. *Sens. Actuators, B* **318**, 127937 (2020).

34. Lu Z, Fan W, Shi X, Black CA, Fan C, Wang F. A highly specific BODIPY-based fluorescent probe for the detection of nerve-agent simulants. *Sens. Actuators, B* **255**, 176-182 (2018).

35. Jang YJ, Mulay SV, Kim Y, Jorayev P, Churchill DG. Nerve agent simulant diethyl chlorophosphate detection using a cyclization reaction approach with high stokes shift system. *New J. Chem.* **41**, 1653-1658 (2017).

36. Jang YJ, Tsay OG, Murale DP, Jeong JA, Segev A, Churchill DG. Novel and selective detection of Tabun mimics. *Chem. Commun.* **50**, 7531-7534 (2014).

37. Singh N, et al. Exploration of fluorescent organotin compounds of alpha-amino acid Schiff bases for the detection of organophosphorous chemical warfare agents: quantification of diethylchlorophosphate. *New J. Chem.* **42**, 8756-8764 (2018).

38. Khan MSJ, Wang Y-W, Senge MO, Peng Y. Sensitive fluorescence on-off probes for the fast detection of a chemical warfare agent mimic. *J. Hazard. Mater.* **342**, 10-19 (2018).

39. Cai Y-C, Li C, Song Q-H. Selective and visual detection of a nerve agent mimic by phosphorylation and protonation of quinolin oximes. *J Mater Chem C* **5**, 7337-7343 (2017).

40. Kim Y, Jang YJ, Lee D, Kim B-S, Churchill DG. Real nerve agent study assessing pyridyl reactivity: Selective fluorogenic and colorimetric detection of Soman and simulant. *Sens. Actuators, B* **238**, 145-149 (2017).

41. Hu X-X, Su Y-T, Ma Y-W, Zhan X-Q, Zheng H, Jiang Y-B. A near infrared colorimetric and fluorometric probe for organophosphorus nerve agent mimics by intramolecular amidation. *Chem. Commun.* **51**, 15118-15121 (2015).

42. Zhou C, et al. Isomerization effect of triphenylamine-acridine derivatives on excited-state modification, photophysical property and electroluminescence performance. *Dyes Pigm.* **146**, 558-566 (2017).

43. Zhang S, et al. Achieving a significantly increased efficiency in nondoped pure blue fluorescent OLED: A Quasi-Equivalent hybridized excited state. *Adv. Funct. Mater.* **25**, 1755-1762 (2015).

44. Fan S, Dennison GH, FitzGerald N, Burn PL, Gentle IR, Shaw PE. Acid is a potential interferent in fluorescent sensing of chemical warfare agent vapors. *Commun Chem* **4**, 45 (2021).

45. Ha S, et al. Structural effect of thioureas on the detection of chemical warfare agent simulants. *ACS Sens.* **2**, 1146-1151 (2017).
